# Adolescents, menstruation, and physical activity: insights from a global scoping review

**DOI:** 10.1186/s12905-025-03825-w

**Published:** 2025-06-06

**Authors:** Jessica Harvey, Max J. Western, Nick P. Townsend, Jessica Francombe-Webb, Simon Sebire, Olivia S. Malkowski, Masha Remskar, Ella Burfitt, Emma Solomon-Moore

**Affiliations:** 1 Department for Health, Centre for Motivation and Behaviour Change, University of Bath, Bath, UK; 2 Centre for Exercise, School for Policy Studies, Nutrition and Health Sciences, University of Bristol, Bristol, UK; 3 Department for Health, Centre for Sport, Physical Activity & Health Equality, University of Bath, Bath, UK; 4 The Health Improvement Commission for Guernsey and Alderney LBG, Castel, Guernsey, Channel Islands UK

**Keywords:** Menstruation, Physical Activity, Puberty, Adolescents, Exercise, Sport, Children’s health, Reproductive health

## Abstract

**Background:**

Adolescent girls tend to be less physically active than boys, a trend that coincides with puberty. Menstruation may act as a barrier to physical activity, which in turn may influence menstrual symptoms. The purpose of this scoping review was to synthesise the global literature on the association between menstruation and physical activity among adolescents.

**Methods:**

A systematic search was conducted across five databases, identifying studies on menstruation and physical activity in adolescents (aged 10–18 years) without date restrictions. Studies not in English, including only athlete populations and focusing solely on premenstrual syndrome were excluded. Titles and abstracts, followed by full texts were screened by two independent reviewers.

**Results:**

Eighty-six studies were included, spanning 33 countries. Thematic synthesis of data from the selected studies suggests a bidirectional relationship in that menstruation may act as a barrier to physical activity due to symptoms, societal stigma and menstrual product access, while physical activity may alleviate symptoms for some. The review highlights variability in study methodologies, with most relying on self-report data.

**Conclusion:**

This review provides insights into the varied experiences of adolescent girls’ physical activity and menstruation, influenced by cultural, social, and resource-related factors. It makes important and timely recommendations for the direction of future research, which should employ longitudinal and mixed methods approaches to better understand the association between menstruation and physical activity in this population and address gaps regarding the mechanisms and magnitude of this relationship.

**Supplementary Information:**

The online version contains supplementary material available at 10.1186/s12905-025-03825-w.

## Background

Physical activity (PA) is associated with many physical and psychological health benefits in adolescents [1, 2]. Global guidelines recommend that adolescents engage in an average of 60-min of moderate-to-vigorous-intensity PA per day across the week, as well as bone and muscle strengthening activities three times a week [3]. However, around 81.0% of adolescents do not meet these guidelines globally, with girls less active than boys (84.7% vs 77.6%) [4]. Furthermore, accelerometery data from 10 countries found a decline in PA by 4.2% in adolescents with each additional year of age, but this was steeper in girls (4.6%) compared to boys (3.7%) [5]. Understanding what impacts girls’ PA behaviours is key to improving participation levels [6].

Puberty is an important, yet often neglected factor in PA research, which causes substantial changes in biology, physical appearance, and social and psychological factors for girls [7]. Girls’ total PA level has been shown to be lower in the later stages of puberty compared to pre-puberty [8, 9]. The onset of menarche is a key development during puberty and tends to occur between the ages of 8 and 16 years [10]. Menstruation is accompanied by physical and psychological symptoms, such as dysmenorrhea (menstrual cramping), heavy bleeding, headaches, backache, fatigue, lack of body confidence, and shame, which can impact on girls’ day-to-day lives, including their PA participation [11, 12].

A review of qualitative studies on girls’ self-perception of PA found that menstruation was described as a barrier to being active due to feelings of self-consciousness and discomfort [13]. Reviews of adolescents’ menstrual experiences have highlighted the negative impact menstruation has on girls’ and women’s lives with some suggestions that this includes PA behaviour [14–20]. However, these reviews did not focus specifically on PA and offered little understanding on the mechanisms by which menstruation influences PA behaviour in adolescents. Furthermore, the relationship between menstruation and PA is not necessarily unidirectional. Reviews exploring the influence PA has on menstrual symptoms mostly indicate that PA is positively associated with reduced prevalence or severity of symptoms but tend to include adults in their syntheses [21–27]. Furthermore, to our knowledge, no review has focussed on menstruation and physical activity behaviour in adolescents only and there is a paucity of research that considers menstruation in relation to girls'PA. Therefore, evidence syntheses of adolescent populations are required to understand the association of PA with menstrual symptoms more specifically. Accordingly, this is the first review that aims to map the existing literature globally on the intersection of experiences of menstruation and PA in adolescents. This review seeks to deepen our understanding on the association between menstruation and PA in girls and identify any gaps in the knowledge base on this topic. Through this, the review will help to inform future research and exploration of menstruation and PA in adolescents.

## Methods

A protocol was developed and registered on the Open Science Framework: https://osf.io/se4rz/ on 10/02/2022. This review was conducted in accordance with Arksey and O’Malley’s [28] scoping review methodology and follows the reporting guideline of the Preferred Reporting Items for Systematic Review and Meta-Analyses extension for Scoping Reviews (PRISMA-ScR) checklist (Additional file 1) [29]. The review aims to answer the following research questions: What is known about the association between menstruation and physical activity in adolescent girls globally and what gaps remain? Which methods have been used to study the association between menstruation and physical activity in this population?

### Information sources

A subject librarian worked with the primary researcher to identify relevant keywords and advised on which databases were most likely to produce the type of studies required and to inform the final search strategy. An initial search was conducted on 03/03/2022 and a second search on 24/09/2024 (to ensure literature was up to date) to identify peer-reviewed literature in the following databases: PubMed, Web of Science, APA PsycNET, Scopus and SportDiscus. The reference lists of included sources of evidence were screened for additional studies. This scoping review considered both experimental and quasi-experimental study designs, as well as observational studies including prospective and retrospective cohort studies, case–control studies, cross-sectional studies and qualitative research.

### Search strategy

The final search strategy used the terms physical activity (AND), menstruation (AND) adolescents including derivatives of these terms (Additional file 2) and pilot tested (using Scopus, PubMed and Web of Science) to assess the search filters and ensure the quality of the final search strategy. The text words contained in the titles and abstracts of relevant articles were used to develop the full search strategy. The search strategy, including all identified keywords and index terms were adapted for each included database.

### Study selection

Following the search, all identified citations were collated and uploaded into Endnote and Covidence, and duplicates removed. The screening process was conducted by a team of five researchers in two stages: first titles and abstracts of all retrieved citations and secondly the full texts of the selected studies were assessed for inclusion against the eligibility criteria (see below). One researcher (JH) independently screened the title and abstract and full text of all retrieved citations. Four additional reviewers (OSM, MR, EB, ESM) divided the retrieved citations between them so that all titles and abstracts, and full texts were reviewed by two people. The reviewer and assistant reviewers piloted screening with 10 articles to ensure the eligibility criteria were clear. Articles deemed relevant by two reviewers were included in the full text review. Any discrepancies between inclusion and exclusion throughout the process (pilot and screening) were discussed in meetings and resolved by consensus amongst the research team.

An additional three studies were identified from the reference lists of eligible papers. The inclusion criteria were guided by the ‘Population-Concept-Context’ framework for scoping reviews [30]. In line with the guidelines of a scoping review [30], the studies were not evaluated for quality and all reporting is based on direct presentation of results from the authors of the included studies. Due to the high volume of relevant academic literature identified by the search, we chose not to include grey literature. Inclusion and exclusion criteria for the scoping review are described in Table 1.

**Table 1:** Inclusion and exclusion criteria

Inclusion criteria:
Participants:
• Adolescents between the ages of 10–18 years. For studies including ages outside of this age range, studies were included if the mean age was ≥ 10 years and < 19 years • Studies must include age-disaggregated data/information on school-aged adolescent ages (10–18 years) • Non-athletes
Concept:
• Studies must include data/information on menstruation/menstrual health*/menstrual cycle and PA
Context:
• All study designs were included • All PA contexts were included (within and outside of school, leisure, sports clubs, active travel) • No restrictions on country/settings • No limitation on dates published
Exclusion criteria:
• Only measured puberty and not menstruation specifically (i.e., did not isolate menstruation from maturation/puberty) • Written in a language other than English owing to the linguistic capabilities of the review team • Sample only includes adults (aged > 18 years). Where studies included ages ranging from 10–19, if the mean age in years was < 19 years the study was included • Case reports, clinical opinion pieces, commentary articles, or if the text did not include the full study report (e.g., conference abstracts without full paper)

*Menstrual health can include reference to dysmenorrhea and other menstrual symptoms

In line with the iterative process of the scoping review methodology, some decisions regarding eligibility were made at the different stages of the reviewing process once authors had familiarised themselves with the literature. For example, it was decided that athletes would be excluded as exercising at very high levels can lead to different menstrual characteristics compared to those participating in moderate or low-level exercise [31]. Secondly, studies which explored premenstrual syndrome (PMS) were not included, however, studies capturing the menstrual experiences of participants with specific menstrual symptoms (e.g., dysmenorrhea) were eligible. Dysmenorrhea and PMS are often considered together within studies but unlike PMS, dysmenorrhea occurs immediately prior to and/or during menstruation itself [32].

### Data extraction and synthesis of results

Data extraction was conducted by the primary researcher guided by a charting form established a priori (Additional file 3) to ensure that all required information was captured consistently. The data extraction form was piloted on a small number of included studies to ensure consistency and completeness before full data extraction was undertaken. Following this, the lead researcher (JH) familiarised themself with the data by reading all included studies and reviewing the charted data. The findings were then mapped to concepts related to the research question and discussed with the wider research team [33]. Concepts included: 1) measurement tools for PA and menstruation, 2) studies reporting on the impact of menstruation and menstrual symptoms on PA behaviour, and 3) studies examining the impact of physical activity on menstrual symptoms. Although the charting form included plans to extract information on theoretical frameworks and policy or practice recommendations, these aspects were not discussed due to insufficient reporting in the included studies.

## Results

### Study characteristics

**Fig. 1:**
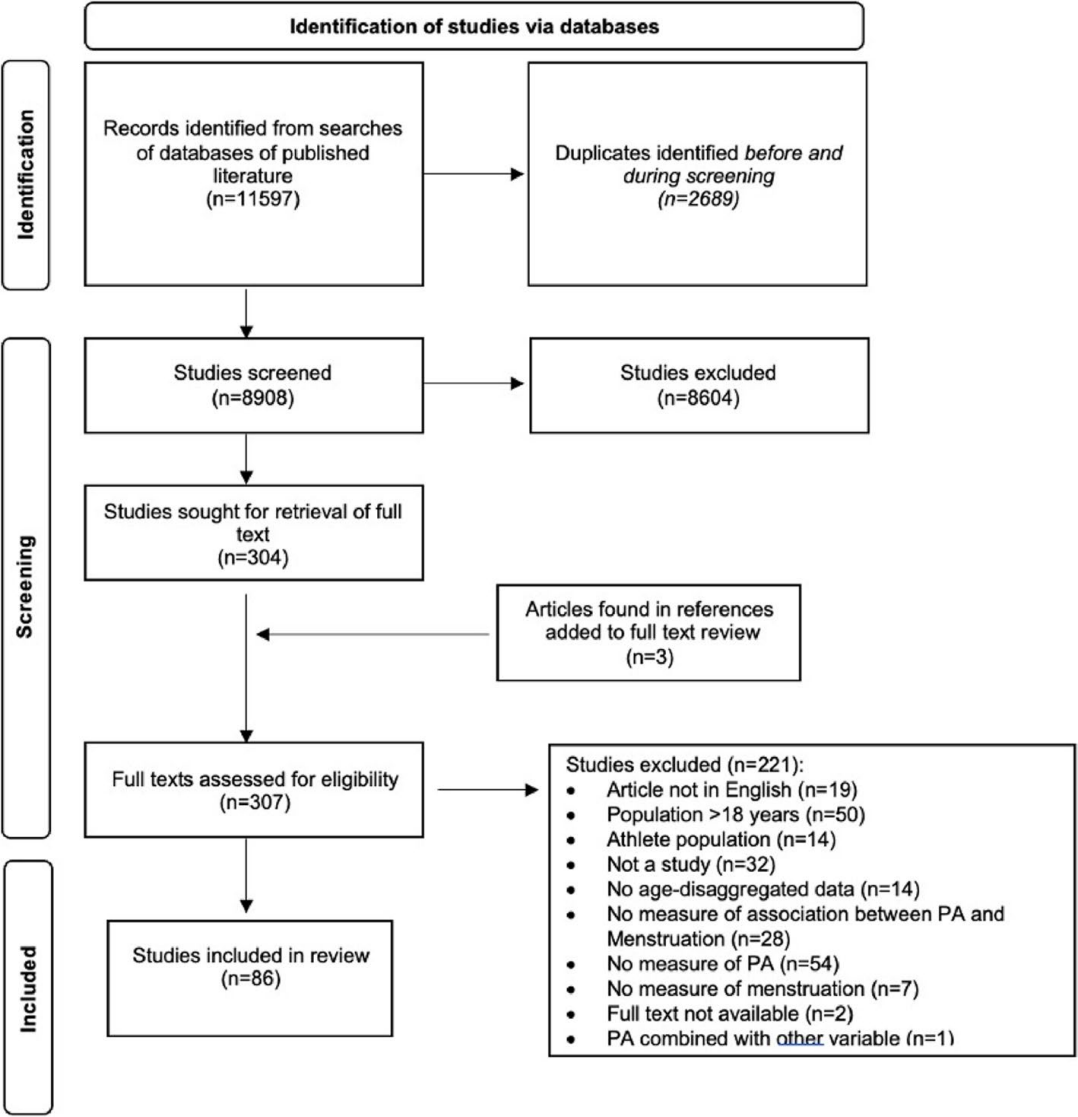
PRISMA-ScR diagram of studies

Results of the search and study inclusion process are presented in Fig. 1 [34]. The scoping review included a total of 86 studies which were published between 1958 and 2024 and conducted in 33 countries, with the most common being in India (*n* = 15) and the USA (*n* = 14). The majority of studies were observational (*n* = 54), 15 were experimental, 14 qualitative, and three mixed methods. Most studies were conducted within a school setting (*n* = 66), eight recruited hospital outpatients, three studies used secondary data from national surveys, five studies recruited from a community setting, three study recruited participants using social media and one from a university. The study sample sizes ranged from 7 to 12,707 participants. Forty-seven studies were conducted in low- or middle-income countries (LMIC), 38 in high-income countries (HIC) and one study was in both [35] (Table 2). Further information about each study can be found in the Additional files 4–7.

**Table 2:** Summary of eighty-six included studies

Study type	Number	Countries									Year range	Context
Cross-sectional	50	Australia (*n* = 4)[36–39] Belgium (*n* = 1)[40] Brazil (*n* = 1) [41] Canada (*n* = 2)[42, 43] Chile (*n* = 1)[44] Egypt (*n* = 1)[45] Finland (*n* = 1)[46]		France (*n* = 2)[47, 48] Germany (*n* = 1)[6] Ghana (*n* = 1)[49] India (*n* = 10)[50–59] Indonesia (*n* = 2)[60, 61] Iran (*n* = 2)[62, 63] Israel (*n* = 1) [64]		Japan (*n* = 1)[65] Malaysia (*n* = 2)[66, 67] Morocco (*n* = 1)[68] New Zealand (*n* = 1)[69] Nigeria (*n* = 2)[70, 71] Pakistan (*n* = 1)[72] Saudi Arabia (*n* = 1)[73]		South Africa (*n* = 1)[74] South Korea (*n* = 2)[75, 76] Sri Lanka (I = 1)[77] Turkey (*n* = 1)[78] Uganda (*n* = 1)[79] USA (*n* = 5)[80–84]			1989–2024	Social media (*n* = 3) Hospital outpatients (*n* = 5) National Population Registry (*n* = 1) National Survey (*n* = 2) Schools (*n* = 36) Community (*n* = 2) University (*n* = 1)
Case control	1	USA[85]									2011	Hospital Outpatients
Prospective	2 1	USA[86, 87] Uganda[88]									1984–2024	Schools (*n* = 2) Community (*n* = 1)
Quasi-experimental	2	Indonesia[89, 90]									2017–2018	Schools
Prospective intervention	1	India[91]									2013	School
Randomised control trial	7	Egypt(*n* = 1)[92]			India (*n* = 1)[93]		Iran (*n* = 4)[94–97]			Saudi Arabia (*n* = 1)[98]	2004–2024	Schools (*n* = 5) Community (*n* = 1) Hospital (*n* = 1)
Pilot intervention	1	Uganda[99]									2020	School
Non-randomised intervention	4	USA [100–103]									1958–1963	Schools
Participatory soma-based	1	Sweden[104]									2021	School
Grounded-theory	1	Australia[105]									2020	Hospital Outpatients
Qualitative	12	Canada (*n* = 1)[106] China (*n* = 1)[107]	Ghana (*n* = 1)[108] India + Canada (*n* = 1)[109]			Kenya (*n* = 1)[110] South Africa (*n* = 1)[111] Taiwan (*n* = 1)[112]			Tanzania (*n* = 2)[113, 114] USA (*n* = 2)[115, 116] UK (*n* = 1) [117]		2006–2024	Schools (*n* = 10) Schools and Online (*n* = 1) Community (*n* = 1)
Mixed methods	3	India (*n* = 2)[118, 119]				Zambia (*n* = 1) [120]					2018–2019	Schools

The following results are structured to address the two research questions guiding this review. First, we summarise the methods used in the included studies. Next, we present findings on how PA activity influences menstruation and menstrual symptoms, including through interventions and coping strategies. Finally, we examine how menstruation may affect PA behaviours among adolescent girls, focusing on barriers, perceptions, and contextual factors. Together, these themes provide a comprehensive overview of the current evidence and highlight remaining gaps in the literature. Studies are discussed within each subsection based on relevance, with the type of study design (qualitative, quantitative, or mixed methods) indicated throughout.

### Measures of menstruation

The most common measures of menstruation (including menstrual symptoms) used in the reviewed studies were menstrual characteristics such as age at menarche, regularity of cycle, menstrual flow, length of cycle, duration of menstruation, and severity of symptoms. Six studies used existing questionnaires to assess menstruation [40, 42, 63, 75, 82, 93]. These included the Menstrual Disorders of Teenagers Questionnaire [121], the Menstrual Distress Questionnaire [122], the Menorrhagia Impact Questionnaire [123] and the Andresh Milsom Scale for dysmenorrhea severity [124]. For the remaining studies it was often unclear whether custom questionnaires were developed for the study, existing tools were adapted, or a combination of both approaches was used. Thirteen studies used a visual analogue scale to measure menstrual pain intensity and or severity, whilst two used a pictorial blood loss assessment chart for blood loss. Other methods for collecting menstruation data included a diary (*n* = 4) and a menstrual calendar (*n* = 1). Of the 14 qualitative studies, eight used focus groups, four conducted semi-structured interviews, and two used combined methods (observations and/or interviews or workshops).

### Measures of PA

In 20 of the observational studies (*n* = 54), PA was measured using self-report or interviewer led questionnaires, while the remaining 33 studies had no measure of PA but asked participants how menstruation or menstrual symptoms influenced PA, sport or exercise participation. For those using self-report measures, PA was measured using a wide range of approaches, most of which were non-standardised and varied in structure and detail. For example, some used simple, dichotomous items asking whether participants engaged in PA or regular exercise (e.g. yes/no) [41, 47, 57, 60, 64, 76]. Several studies assessed frequency of activity, such as the number of days per week or month participants were active [6, 46, 50, 52, 54, 75, 125] or the regularity of sports participation [69]. Two asked participants to estimate time spent being active (e.g. hours per day/week) [65, 70]. Only two studies used more structured or validated tools, the International Physical Activity Questionnaire [61] and the Health-Promotion Lifestyle Profile Questionnaire [63]. One study aligned its question with WHO PA guidelines by asking if participants engaged in ≥ 30 min of activity on five or more days per week [73]. Another asked about types of sport played over the past 12 months [86]. Overall, the heterogeneity and lack of standardisation in self-report measures limited comparability across studies and highlights the need for more consistent and validated approaches to measuring PA in this population.

Physical activity was used as a symptom management intervention in 11 experimental studies, nine of which provided no data on adherence, while a further two experimental studies examined the influence of educational interventions on self-reported PA. Four qualitative studies explicitly asked about PA [106, 108, 109, 117], with the remaining 10 including PA as a participant reported theme in their analysis. No study measured PA objectively (e.g., via accelerometery).

### The influence of PA on menstrual symptoms

Twenty-two observational studies investigated whether PA was associated with menstruation and menstrual symptoms. Eight of which found PA was positively associated with fewer or less severe menstrual symptoms, i.e., more active girls were less likely to suffer with dysmenorrhea [52, 60, 61, 64], have severe pain [46, 50, 63], or report menstrual problems [57]. Some of the observational findings suggested that the association between PA and dysmenorrhea was influenced by symptom severity or PA intensity. For example, Kazama and Maruyama [65] reported that higher levels of sport club activities were associated with lower prevalence of severe dysmenorrhea. Another study found that mean pain score for dysmenorrhea was higher in girls who reported being unable to participate in sports compared to those who reported continuing with normal activity during menstruation [67]. While another found that participants who do not participate in moderate-vigorous intensity PA and/or only light-intensity PA were more likely to experience dysmenorrhea than those who participate in moderate- or vigorous-intensity PA [73]. Findings regarding other menstrual symptoms were unclear. Two observational studies demonstrated that lower levels of PA were associated with irregular menstruation [52, 76]. Shinde and Laddad [52] also reported that a greater proportion of girls (19.44%) who did PA on less than three days per week suffered with menorrhagia (heavy menstrual bleeding [HMB] or bleeding for > 7 days) than those who were active daily (13.25%). In contrast, findings from a qualitative study conducted in China revealed participants felt being active made their period heavier and increased severity of pain [107]. However, seven cross-sectional quantitative studies found no evidence that PA was associated with menstruation or menstrual symptoms in adolescents [47, 55, 66, 68, 75, 77, 80]. Furthermore, the one longitudinal study reviewed found no correlation between dysmenorrhea, menstrual regularity or duration of menstruation with exercise, with the exception of two sports [86]. In this study, participation in dance in the previous 12 months was associated with irregular menses, while swimming participation in the previous 12 months was associated with regular menses but an abnormal duration of flow compared to those taking part in other sports [86].

Findings on whether participants used PA to manage menstruation or menstrual symptoms were mixed. Eight cross-sectional studies found that girls reported using exercise to manage or reduce menstrual symptoms [42, 45, 47, 49, 64, 71, 77, 81]. This was consistent with findings from two qualitative studies reviewed where girls described enjoying doing sport or moving to reduce menstrual cramps [104, 114]. However, the prevalence of participants reporting using exercise to manage symptoms in the cross-sectional studies ranged from 4.8% to 57%, and other pain-relief techniques were more widely reported in three studies which included medication, heat, and rest [64, 77, 114]. Furthermore, one cross-sectional study conducted in Iran, found that 66% of participants did not believe in the effectiveness of PA to reduce menstrual pain [62].

#### Exercise interventions for menstrual symptoms

Thirteen studies investigated the effect of an exercise intervention on dysmenorrhea (see supplementary file 5 for details of each study) [89–91, 93–95, 97, 98, 100, 102, 103, 126, 127]. Six were randomised control trials (RCT) [93–95, 97, 98, 127] and the remaining seven were non-randomised intervention studies. The interventions varied greatly in design and included stretching exercises [89, 94, 97, 100, 102, 103, 126], yoga [91], light intensity exercise with stretching [95], mild aerobic exercise [98] and aerobic and strength exercise vs aquatic exercise [127], while two did not report on the type of PA intervention used [90, 93]. The interventions had an average duration of six months (ranging from two months to three years). However, due to methodological heterogeneity of interventions, it was not possible to make comparisons based on intervention duration. All studies reported that the intervention reduced the severity or prevalence of dysmenorrhea. One study also reported a reduction in other menstrual symptoms such as backache, lower limb pain, irritability and lack of concentration following a six-month yoga intervention [91].

### The role of menstruation on PA levels

Fourteen studies investigated the role menstruation plays in influencing PA behaviour. Twelve cross-sectional studies [36, 40, 48, 51, 53, 56, 59, 62, 72, 74, 78, 84] and two mixed-method studies [118, 120] found that menstruation had a negative association with PA, where girls reported avoiding or restricting PA due to menstruation. The prevalence of participants reporting restricting their PA behaviour varied widely across the studies, ranging from 25.2% to 61.1%. Only one of these studies explored the reasons participants gave for restricting activity which included physical symptoms, fear of swimming, embarrassment, perceived inability to perform, desire to abstain from activities, perceived increase in bleeding, and difficulties in managing menstruation [74].

Findings from a further fourteen observational (13 cross-sectional and one prospective) studies indicated that girls reported avoiding or restricting PA due to specific menstrual symptoms, most commonly dysmenorrhea [37, 41, 45, 47, 49, 50, 58, 69, 77, 81, 128], but also HMB [44, 82], and having irregular menstruation [6]. Secondly, those who experienced more severe menstrual pain were more likely to limit PA participation compared to those with mild pain [36, 45, 50, 81, 128]. Torres and Zajer [44] found in their study that 80.4% of participants who experienced HMB reported missing PE in school due to this symptom.

These results coincide with the findings of the qualitative studies that reported some girls avoid PA during menstruation, and participants restrict or avoid PA due to fear of exposure and concerns around leaking due to menstruation [105–107, 111, 112, 115–117]. In two studies, girls described how menstrual pain and symptoms such as low mood, breast pain, fatigue and nausea restricted movements, making PA challenging [104, 114, 117]. Other reasons leading to avoidance of PA during menstruation included lack of energy and feeling uncomfortable [113, 106].

### Resources/setting

#### Period products

Use of period products and the role they play in PA behaviour was discussed in four of the qualitative studies. Some participants described using specific products such as tampons to help manage menstruation when being active, notably for swimming [83]. Others mentioned avoiding certain activities (e.g., cheerleading, gymnastics) if it meant needing to wear a tampon [115, 116]. Li and colleagues [105] reported that some participants described feeling too young to use tampons and they would avoid swimming during menstruation for fear of leaking.

Access to menstrual products was described as a barrier to PA in four qualitative studies [108, 110, 113, 114] and one mixed-method study (cross-sectional and qualitative) conducted in LMICs [118]. Lack of access to period products meant that girls found it difficult to engage in normal activities such as running, walking and playing during their period [110, 114]. The type of menstrual product used was associated with how active girls were, where those using reusable pads were less likely to report avoiding PA compared to those using cloth or toilet paper. Moreover, inadequate materials or facilities to manage menstruation reportedly caused discomfort to participants during long walks to school [113], and girls found it challenging to actively commute to [108] or even attend school [118] without adequate period products due to the risk of leaking. However, one cross-sectional study in Canada, examining the impact of menstrual poverty on sports also found that 40% of participants reported missing sports or gym during menstruation due to lack of money for period products [43].

#### Clothing/resources

Clothing was also raised as a possible barrier to being active during menstruation in three of the qualitative studies reviewed [113, 115, 117]. Participants explained that tight fitting clothing and shorts created anxiety around possible exposure, which made it more challenging to be active during menstruation [115]. Secondly, participants in another study described preferring darker coloured clothing for PA [117]. Both material resources and the social context in which participants experience menstruation appeared to play a role in PA behaviour. One study in a LMIC found that participants in schools with adequate resources for PA, sanitation facilities and access to menstrual products, were more likely to restrict sporting activities at school whilst menstruating, compared to those in less resourced schools [74]. Furthermore, this study found participants in more resourced schools reported physical symptoms as the most common reason for not participating in sporting activities, compared to those in under-resourced schools who reported menstrual management problems and fear of discovery as barriers to PA.

#### Education

Four of the studies reviewed explored menstrual education. Two intervention studies conducted in LMICs aimed to improve reproductive and menstrual health in adolescents. One compared the effects of different training delivery methods (using parents or school health trainers) on reproductive health promotion [96]. They found when training was provided by school health trainers, girls were more likely to exercise during menstruation, compared to when training was provided by parents. Another study piloted a multi-component school-based intervention aiming to improve menstrual health and hygiene and school attendance [99]. The intervention was associated with a reduction in the proportion of girls who reported avoiding PA during menstruation. Results of the qualitative component of this study suggested that girls learned to use reusable pads to manage menstruation during PA and exercise to relieve menstrual pain through the intervention.

One cross-sectional study reviewed included an open-ended question about menstrual education where participants reported the menstrual education they received lacked applicability to their everyday activities, e.g., sports class [39]. Furthermore, a qualitative study in the UK identified an absence of education on how to exercise during menstruation as a barrier to girls being active [117].

### Attitudes and perceptions towards menstruation and PA

#### Beliefs

Differences between countries in their cultural and contextual beliefs towards being active during menstruation was highlighted in this review. The qualitative findings from two studies conducted in India found that girls believed they should not be active whilst menstruating and therefore avoided taking part in activities which involved physical exertion during this time [109, 119]. This was information passed onto them by family members, namely mothers, and something they themselves did not understand. In contrast, a qualitative study in the US found that some girls were encouraged by their households to use exercise to manage menstrual symptoms, instead of using painkillers to reduce pain [115]. Other girls in this study viewed menstruation as ‘dirty’ and that being active in school would require ‘multiple showers’ and, therefore, they refrained from taking part in PE in school [115]. A longitudinal study in Uganda found an association between menstrual anxiety and PA, whereby girls who avoided PA during menstruation were more likely to report anxiety about the next period [88]. Anxiety about the next period was more common among those who agreed it was unhealthy for a girl to run, dance or cycle during her period.

#### Stigma

Feeling shame and embarrassment during menstruation was discussed in four of the qualitative studies conducted in HICs. Leaking menstrual blood was a common concern and two studies revealed that girls would prefer to sit out of activities to avoid risk of embarrassment [115, 117]. Concerns about informing male teachers that they could not participate in sports, such as swimming, due to being on their period was also reported [105]. Finally, feeling a lack of understanding from coaches, particularly male coaches, led to frustrations for some girls whilst playing sport during menstruation [104].

## Discussion

The aim of this scoping review was to synthesise literature on the association between menstruation and PA in adolescents. Despite the large variability of study methodologies and mixed findings, the results of this review indicate a potential bidirectional relationship between menstruation and girls’ PA behaviour. The findings suggest girls’ experiences of menstruation and PA are varied, in that some may avoid or restrict PA due to menstrual-related barriers, while others perceive and use PA as a possible way to manage symptoms. Although the findings demonstrate a complex relationship between PA and menstrual symptoms, PA may reduce the severity or prevalence of menstrual symptoms, namely dysmenorrhea. The review reveals that menstruation is accompanied by factors which may create barriers to PA for girls, such as practical challenges (e.g., lack of period products, clothing) and psychosocial issues (e.g., stigma, lack of education). The variability in the findings might be due to the different study designs and contexts as results varied between LMICs and HICs as well as the complexity of the bio-psycho-social experiences of menstruation. This suggests that when seeking to understand adolescents’ experiences of menstruation it is not only important to focus on the physical environment, material resources including sanitation, and infrastructure, but also the social and cultural context.

The findings suggest that adolescents frequently view menstruation and menstrual symptoms as having a negative impact on their PA behaviour. A common theme in the studies reviewed was that girls reported avoiding or restricting PA due to menstruation. These findings are consistent with previous reviews exploring menstrual experiences, where participants also reported restricting PA due to menstruation [14, 15, 17, 27]. Similarly, in a review of girls’ PA behaviour, it was found that participants reported menstruation as a barrier to them being active [13]. Efforts to intervene or provide PA opportunities for adolescent girls may need to consider menstruation as a factor in behaviour change.

The findings from both observational and intervention studies included in this scoping review are similar to those of previous reviews, explicitly exploring the role of PA in mitigating dysmenorrhea in adolescents and adults. These showed that PA reduced dysmenorrhea symptoms and severity [22, 23]. However, the interventions reviewed varied greatly in design preventing meaningful comparison between studies. Previous reviews found similar challenges in drawing conclusions due to high heterogeneity of included studies. This highlights a need for studies to better report the characteristics of their PA interventions, including measures of PA (dose, frequency, intensity, duration and type) and menstrual symptoms, to accurately determine the role of PA in reducing menstrual symptoms in this population.

Findings regarding the role of PA on other menstrual symptoms were mixed and unclear, similar to those from previous reviews. Armour and colleagues [19] found limited primary studies measuring this association and could not draw firm conclusions due to conflicting results. Daley [32] reported that PA was associated with reduced prevalence of menstrual symptomatology in the observational studies and one RCT reviewed. However, the menstrual symptoms were not defined. These findings demonstrate a potentially complex relationship between PA and menstrual symptoms, which may be confounded by a range of other factors (e.g., other menstrual management strategies). Within this review, studies which investigated whether girls used PA to manage menstrual symptoms reported that exercise was perceived to relieve symptoms, yet notably, other pain-relief techniques were also mentioned, such as medication, heat, and rest. A recent review examining self-care strategies for menstruation found that one in ten young women used exercise to manage menstrual symptoms, but rest was the most common non-pharmacological self-management strategy used [129]. However, the effectiveness of these self-management strategies could not be determined due to a lack of primary studies looking at this association. Further research to understand the protective role of PA during menstruation is needed.

Period products appeared to play a role in the PA behaviour of some participants. This was similar to the findings of a review of 104 studies in 16 countries aiming to explore menstrual health in HICs [14]. They reported that some menstrual practices (use of period products to contain menstrual bleeding), influenced participants’ confidence or choice of whether to engage in sporting activities (e.g., swimming), with many individuals avoiding PA during menstruation. Secondly, the findings from the current review indicate that limited access to period products is a potential barrier to PA among adolescent girls across diverse settings. In LMICs, this barrier was primarily linked to product availability, while in HICs, affordability was a more common concern. Improving both access to and education about period products may help reduce this barrier to participation in PA. For example, an intervention aiming to improve menstrual health in LMICs was effective in supporting girls to engage in more activities during menstruation [130]. This study found that, when provided with new period products alongside educational information, girls felt they could ‘*contain’* their menstruation and engage in activities they had not previously considered doing during menstruation, such as playing sports. Education was also highlighted as a barrier to PA in a review on experiences and perceptions of the menstrual cycle conducted in women where a need for PA guidelines for individuals who menstruate was suggested [27].

The results suggest that there may be different barriers experienced by girls in LMICs and HICs due to resource availability, sanitation and infrastructure. However, despite adequate provision of facilities and products and available information, menstruation is often considered embarrassing and shameful. For example, concerns around period products and leaking links to an important, yet understudied, finding from this review regarding the stigma felt by some girls around menstruation. This is similar to previous reviews in both LMICs and HICs that demonstrate the influence of menstrual stigma on confidence to engage in PA during menstruation due to feelings of shame and embarrassment [14, 15, 17, 27]. Furthermore, studies conducted in athlete populations suggest these common concerns of embarrassment and shame around menstruation track through into adulthood [131]. This highlights an important area for research on girls’ PA behaviour and efforts should be made towards dismantling cultures of shame, stigma and misinformation.

### Limitations of reviewed studies

This review has highlighted several gaps in the literature. Firstly, many of the studies included self-reported measures of PA with little information about their reliability and validity, meaning a precise assessment of behaviour is lacking in the evidence base. Future studies should seek to use validated device-based measures of PA such as accelerometery [132, 133] for a more accurate representation of behavioural patterns, intensity and volume and its reciprocal relationship with menstruation. Additionally, no studies measured PA for a full monthly cycle to determine whether and/or how physical activity behaviour changes during menstruation.

Secondly, studies tended to use retrospective methods to measure menstruation. Typically, participants were asked to recall symptoms or experiences from either their most recent cycle or up to a year before, leading to potential recall bias. Participants tend to recall pain experienced most severely or closest to completing a questionnaire [131]. Furthermore, many menstrual symptoms were not measured or considered in the studies reviewed. The most common symptom examined was dysmenorrhea, but menstruation is accompanied by many others, such as heavy bleeding, headaches, backache and fatigue [134]. Studies using simultaneous objective measures of PA and menstrual symptoms may provide more insight into the relationship between PA and menstruation in adolescents.

Amongst the observational studies reviewed, there was no empirical focus on the possible bi-directional associations between menstruation and PA, thus precluding any causal inferences. For example, it is not possible to determine whether the results of the cross-sectional studies on menstrual pain and PA are because girls with higher pain scores participated in less PA due to pain or whether PA reduced pain. Longitudinal study designs are important for temporal associations and could also be used to understand the possible bi-directional associations between PA and menstruation. A longitudinal study using daily measurements of PA and menstruation may provide more timely assessments of fluctuations in PA and menstrual symptoms.

Only three mixed-methods studies were identified in this scoping review. Mixed-method approaches may be beneficial when looking to understand menstruation and PA as qualitative findings describe why there may be some diversity in the strength and direction of a possible association. Finally, the qualitative studies’ aims were not focussed on PA and menstruation and, therefore, lacked the depth of understanding that a study delving into this topic specifically might gain. Table 3 summarises the key research and policy and practice recommendations in this review.

**Table 3:** Summary of research and policy recommendations

Recommendations
Research • Use validated device-based measures for accurate assessment of physical activity • Monitor physical activity across the full menstrual cycle • Measure a wider range of menstrual symptoms beyond dysmenorrhea (e.g. heavy bleeding, fatigue, headaches, back pain) • Apply real-time or momentary assessment methods to track menstrual characteristics and experiences • Conduct longitudinal studies to explore temporal and bi-directional associations between menstruation and PA • Incorporate mixed methods to capture individual and contextual influences • Undertake qualitative research focused specifically on menstruation and PA • Conduct high-quality RCTs to evaluate exercise as a treatment for dysmenorrhea
Policy and Practice • Integrate comprehensive, evidence-based menstrual and physical activity education into school curriculum • Improve access to and affordability of period products for all adolescents • Foster supportive, non-stigmatizing environments around menstruation in schools and sports settings • Ensure access to, and choice of, suitable clothing options for physical activity during menstruation • Educate adolescents on the potential benefits of PA for menstrual symptoms

### Strengths and limitations of this scoping review

To our knowledge, at the time of publication, this is the first review to explore the literature on menstruation and PA in adolescents. Effort was made to minimise bias throughout the review process and four reviewers assessed the eligibility of included studies. The search strategy included five electronic databases identifying peer-reviewed literature. However, grey literature was not included in the search for this review due to feasibility constraints due to having a small research team and the large number of citations retrieved in the abstract screening. For practical reasons, we were not able to include studies that were in languages other than English and therefore further eligible studies may have been missed. Furthermore, four of the studies were conducted between 1958 and 1968 [100, 102, 103, 126], making comparisons with more modern contexts, cultures, interventions, and data collection methods challenging. For example, advertisement of menstrual products in the 1950 s reinforced the idea that menstruation should be hidden and brands historically have used blue dye to depict menstrual blood [135, 136]. However, more recently brands have started to use red dye to depict menstrual blood and have been criticised for using words such as ‘discrete’ to discuss menstrual products, demonstrating a cultural shift on this topic [137]. Including studies conducted in different time periods will impact our findings due to changes in the discourse and attitudes around menstruation over more recent decades.

## Conclusion

This scoping review highlights the complex and possible bi-directional association between menstruation and PA in adolescent girls. The findings suggest that menstruation can be a barrier to girls’ PA due to menstrual symptoms, stigma, lack of education and practical challenges such as clothing rules and access to period products. However, PA may have potential benefits including reducing the severity of dysmenorrhea with some adolescents reportedly using PA as a symptom-management strategy. The review also identified differences between LMICs and HICs. In LMICs, barriers commonly stemmed from limited access to menstrual products and inadequate sanitation facilities, while in HICs, psychosocial factors such as stigma and embarrassment were more prominent. This emphasises the importance of context-specific approaches to addressing menstruation and PA. Future research could adopt longitudinal and mixed method designs to determine the temporal associations between menstruation and PA in this population. Utilising objective measures of PA and menstrual symptoms may provide a deeper understanding and insight into this topic alongside qualitative exploration of menstruation as a bio-psycho-social lived experience. Furthermore, interventions which address practical barriers, reduce stigma and promote menstrual health education amongst a range of stakeholders, including girls, may help improve adolescent girls’ PA participation during menstruation and more generally.

## Supplementary Information

Additional file 1. PRISMA-Scr Checklist. PRISMA-Scr Checklist. 20-item checklist for reporting scoping reviews.

Additional file 2. Search Strategy. Search Strategy. Summary of full search strategy.

Additional file 3. Charting Tool. Table of charting tool for data extraction. Charting Tool for data extraction.

Additional file 4. Qualitative studies included in review. Table of fourteen qualitative studies included in the scoping review. Table displaying summary and description of qualitative studies included in the scoping review.

Additional file 5. Intervention studies included in review. Table of fifteen intervention studies included in the scoping review. Table displaying summary and description of intervention studies included in the scoping review.

Additional file 6. Observational studies included in review. Table of forty-three observational studies included in the scoping review. Table displaying summary and description of observational studies included in the scoping review.

Additional file 7. Mixed-methods studies included in review. Table of three mixed-methods studies included in the scoping review. Table displaying summary and description of mixed-methods studies included in the scoping review.

## Data Availability

The datasets used and/or analysed during the current study are available from the corresponding author on reasonable request.

## Abbreviations

PA: Physical Activity
PE: Physical Education
PMS: Premenstrual Syndrome
PRISMA-ScR: Preferred Reporting Items for Systematic Review and Meta-Analyses extension for Scoping Reviews
UK: United Kingdom
LMIC: Low-middle-income countries
HIC: High-income countries
HMB: Heavy Menstrual Bleeding
RCT: Randomised Control Trial
