# Supplementary material for: Adolescents, menstruation, and physical activity: insights from a global scoping review
Source: BMC Womens Health. 2025 Jun 6;25:281. doi: 10.1186/s12905-025-03825-w (PMC12142975; doi:10.1186/s12905-025-03825-w)
Supplement: Supplementary file 2 — Additional file 2. Search Strategy. Search Strategy. Summary of full search strategy. [file 12905_2025_3825_MOESM2_ESM.docx]

**Search Strategy**

1^st^ Search: Conducted 03/03/2022

| **Name of the Database** | **PubMed** |  | **Search Query** |
| --- | --- | --- | --- |
| **Platform** |  | **1** | ("Physical Activity" OR Exercise OR Sport OR "Physical Education") |
| **Year Coverage** | **No limit** | **2** | (Menstruat* OR Menstrual OR Menses OR Menarche OR Dysmenorrhea) |
| **Date of Search** | **03/03/2022** | **3** | (Adolescents OR Adolescence OR Teen* OR "Young People" OR Girls OR Youth OR Children OR School) |
| **Total # of Results** | **1522** | **4** | Filters applied: Adolescent: 13-18 years, Child: 6-12 years |
|  |  |  |  |
| **Name of the Database** | **Web of Science** |  | **Search Query** |
| **Platform** |  | **1** | ("Physical Activity" OR Exercise OR Sport OR "Physical Education") |
| **Year Coverage** | **No limit** | **2** | (Menstruat* OR Menstrual OR Menses OR Menarche OR Dysmenorrhea) |
| **Date of Search** | **03/03/2022** | **3** | (Adolescents OR Adolescence OR Teen* OR "Young People" OR Girls OR Youth OR Children OR School) |
| **Total # of Results** | **2750** | **4** | Filters applied: Adolescent: 13-18 years, Child: 6-12 years |
|  |  |  |  |
| **Name of the Database** | **Scopus** |  | **Search Query** |
| **Platform** | - | **1** | Physical Activity' OR Exercise OR Sport OR 'Physical Education' |
| **Year Coverage** | **No limit** | **2** | Menstruat* OR Menstrual OR Menses OR Menarche OR Dysmenorrhea |
|  | **03/03/2022** | **3** | Adolescents OR Adolescence OR Teen* OR 'Young People' OR Girls OR Youth OR Children OR School |
| **Total # of Results** | **1949** | **4** |  |
|  |  |  |  |
| **Name of the Database** | **APA PsychNet/INFO** |  | **Search Query** |
| **Platform** |  | **1** | Physical Activity' OR Exercise OR Sport OR 'Physical Education' |
| **Year Coverage** | **No limit** | **2** | Menstruat* OR Menstrual OR Menses OR Menarche OR Dysmenorrhea |
| **Date of Search** | **03/03/2022** | **3** | Adolescents OR Adolescence OR Teen* OR 'Young People' OR Girls OR Youth OR Children OR School |
| **Total # of Results** | **411** | **4** |  |
|  |  |  |  |
|  |  |  |  |
|  |  |  |  |
| **Name of the Database** | **SportDiscuss** |  | **Search Query** |
| **Platform** |  | **1** | Physical Activity' OR Exercise OR Sport OR 'Physical Education' |
| **Year Coverage** | **No limit** | **2** | Menstruat* OR Menstrual OR Menses OR Menarche OR Dysmenorrhea |
| **Date of Search** | **03/03/2022** | **3** | Adolescents OR Adolescence OR Teen* OR 'Young People' OR Girls OR Youth OR Children OR School |
| **Total # of Results** | **4003** | **4** | Limited language to English |

2^nd^ Search: Conducted 24/09/2024

| **Name of the Database** | **PubMed** |  | **Search Query** |
| --- | --- | --- | --- |
| **Platform** |  | **1** | ("Physical Activity" OR Exercise OR Sport OR "Physical Education") |
| **Year Coverage** | **04/03/2022** | **2** | (Menstruat* OR Menstrual OR Menses OR Menarche OR Dysmenorrhea) |
| **Date of Search** | **24/09/2024** | **3** | (Adolescents OR Adolescence OR Teen* OR "Young People" OR Girls OR Youth OR Children OR School) |
| **Total # of Results** | **123** | **4** | Filters applied: Adolescent: 13-18 years, Child: 6-12 years |
|  |  |  |  |
| **Name of the Database** | **Web of Science** |  | **Search Query** |
| **Platform** |  | **1** | ("Physical Activity" OR Exercise OR Sport OR "Physical Education") |
| **Year Coverage** | **04/03/2022** | **2** | (Menstruat* OR Menstrual OR Menses OR Menarche OR Dysmenorrhea) |
| **Date of Search** | **24/09/2024** | **3** | (Adolescents OR Adolescence OR Teen* OR "Young People" OR Girls OR Youth OR Children OR School) |
| **Total # of Results** | **569** | **4** | Filters applied: Adolescent: 13-18 years, Child: 6-12 years |
|  |  |  |  |
| **Name of the Database** | **Scopus** |  | **Search Query** |
| **Platform** | - | **1** | Physical Activity' OR Exercise OR Sport OR 'Physical Education' |
| **Year Coverage** | **04/03/2022** | **2** | Menstruat* OR Menstrual OR Menses OR Menarche OR Dysmenorrhea |
| **Date of Search** | **24/09/2024** | **3** | Adolescents OR Adolescence OR Teen* OR 'Young People' OR Girls OR Youth OR Children OR School |
| **Total # of Results** | **72** | **4** |  |
|  |  |  |  |
| **Name of the Database** | **APA PsychNet/INFO** |  | **Search Query** |
| **Platform** |  | **1** | Physical Activity' OR Exercise OR Sport OR 'Physical Education' |
| **Year Coverage** | **04/03/2022** | **2** | Menstruat* OR Menstrual OR Menses OR Menarche OR Dysmenorrhea |
| **Date of Search** | **24/09/2024** | **3** | Adolescents OR Adolescence OR Teen* OR 'Young People' OR Girls OR Youth OR Children OR School |
| **Total # of Results** | **64** | **4** |  |
|  |  |  |  |
|  |  |  |  |
|  |  |  |  |
| **Name of the Database** | **SportDiscuss** |  | **Search Query** |
| **Platform** |  | **1** | Physical Activity' OR Exercise OR Sport OR 'Physical Education' |
| **Year Coverage** | **04/03/2022** | **2** | Menstruat* OR Menstrual OR Menses OR Menarche OR Dysmenorrhea |
| **Date of Search** | **24/09/2024** | **3** | Adolescents OR Adolescence OR Teen* OR 'Young People' OR Girls OR Youth OR Children OR School |
| **Total # of Results** | **132** | **4** | Limited language to English |
