# Supplementary material for: Adolescents, menstruation, and physical activity: insights from a global scoping review
Source: BMC Womens Health. 2025 Jun 6;25:281. doi: 10.1186/s12905-025-03825-w (PMC12142975; doi:10.1186/s12905-025-03825-w)
Supplement: Supplementary file 3 — Additional file 3. Charting Tool. Table of charting tool for data extraction. Charting Tool for data extraction. [file 12905_2025_3825_MOESM3_ESM.docx]

Table of charting tool for data extraction

| Element | Associated Question |
| --- | --- |
| Author | Who wrote the study/document? |
| Year of publication | What year was the document published? |
| Country | Which country is the study/document focussing on? |
| Aim | What are the study/document’s aims? |
| Methodological Design | What is the design of the study? |
| Sample Size | How large is the sample size in the study/document (where relevant)? |
| Population | Who is the target population of the study/document? |
| Theoretical framework used | Did the study use a theoretical framework to inform their research? |
| Measures of PA | How was physical activity measured or recorded in the study/document (e.g., self-report of device-based)? |
| Measures of Menstruation | How was menstruation measured or recorded in the study/document? |
| Setting/Context | Where is the study/document set (i.e. school, sports clubs etc…)? |
| Results/Findings: | What were the most relevant and important findings/results reported in the study/document? |
| *Quantitative results* | Did the study/document report results on the association between menstruation and PA? |
| *Qualitative results* | Did the study/document report any themes/sub-themes on adolescents’ experiences of menstruation and PA? E.g., perceptions, barriers etc… |
| *Practice Recommendations* | Did the study/document make recommendations for PA practices e.g., recommendations for girls PA or for schools? or suggest ways in which adolescents could be supported to be active during menstruation? |
| *Policy Recommendations* | Did the study/document make recommendations for policy? |
| *Research Recommendations* | Did the study report/identify any future research recommendations? E.g., identify gaps in the literature |
| *Other Important findings* | Relevant and useful findings that do not fit into the above outcomes |
