# Supplementary material for: Adolescents, menstruation, and physical activity: insights from a global scoping review
Source: BMC Womens Health. 2025 Jun 6;25:281. doi: 10.1186/s12905-025-03825-w (PMC12142975; doi:10.1186/s12905-025-03825-w)
Supplement: Supplementary file 4 — Additional file 4. Qualitative studies included in review. Table of fourteen qualitative studies included in the scoping review. Table displaying summary and description of qualitative studies included in the scoping review. [file 12905_2025_3825_MOESM4_ESM.docx]

Table of fourteen qualitative studies included in the scoping review

| Author/  Year | Country | Aim/Purpose | Research Design | Sample | Measure of PA | Measures of Menstruation | Context | Main Findings | Quotes |
| --- | --- | --- | --- | --- | --- | --- | --- | --- | --- |
| Brown (2024)(1) | UK | To understand young people’s perceptions  and experiences of menstrual education in schools and their experiences of menstruating whilst at school, including within Physical Education | Qualitative Study | n=48 | Focus Groups | Focus Groups | Schools | Participants reported the complete absence of any education on how to manage periods during PE.  Girls reported feeling nervous about leaking and use extra precautions for being active.  Participants expressed desired changes in PE, linked to activities and exercises.  Uniform and PE kit were discussed across the focus groups with mixed experiences; dark uniform was preferred and there were negative experiences of skirts for PE kit. | "They don’t [provide information], they like you to deal with it [menstrual cycle symptoms] and just get on with it [PE] (Yr 10)"  "I spoke to some other older girls…and some were like “we will use extra, so we’ll use a tampon and a pad when we’re doing exercise just to be doubly cautious” (Yr 8)"  "Rather than saying sit out of PE they [teachers] can give the girls who are on their periods like time to do exercise that helps with cramps or reduces pain. (Yr 9-10)"  "I really liked when the leggings first came out because it makes me feel more comfortable when I’m doing PE because if you leak, it’s not really noticeable compared to when you’re wearing a skirt or shorts. (Yr 8)" |
| Chang (2008) (2) | Taiwan | To explore Taiwanese girls’ experiences of menarche and menstruation and identify key issues for health education | Qualitative Study | n=20 | NA | Focus Groups | School | Menstrual symptoms interfered with physical activities due to fear of leaking and pain. | "I find periods very inconvenient for me. If I have physical education class I will tell my PE teacher that I am uncomfortable and cannot attend the class." |
| Cherenack (2023)(3) | Tanzania | To describe adolescent girls’ experiences of dysmenorrhea and identify sociocultural barriers to dysmenorrhea management in Moshi, Tanzania. | Qualitative study | n=10 | NA | In-depth interviews | Community (including schools and churches) | Three girls described using physical activity as a dysmenorrhea management strategy (Rest, drinking water were more commonly reported) -  Despite the use of physical activity among some girls, other girls had to decrease physical activity during menstruation because of physical symptoms (pain, fatigue, nausea) or worries about leaks due to a lack of menstrual supplies, such as absorbent materials and underwear. | "I find my fellow girls are playing a very good game, but I cannot join in because I am in my period and because they are jumping around, and I cannot do that. It makes me sad, and I hate it" – Adolescent (17, rural setting, in school) |
| Dolan (2014) (4) | Ghana | To assess the impact of sanitary care on the school attendance of girls, as well as the implications of menarche for their well-being | Qualitative Study | n=99 interviews n=135 focus groups | Focus Groups and semi-structured interviews | Focus Groups and semi-structured interviews | School Other: School dropouts | Majority of girls (61.9%) from rural villages said that menstruation causes them to PA compared to 38.4% of those from peri-urban villages  Period product access caused challenges to managing menstruation whilst walking long distances to school, playing and household chores e.g., girls sometimes only used cloth or tissues to manage bleeding and some only had one school uniform | NA |
| Dwyer (2006) (5) | Canada | To explore perceived barriers to participation in physical activity among adolescent girls who live in a large ethnoracially and socioeconomically diverse city | Qualitative Study | n=73 | Structured focus groups | NA | School | Menstruation made it challenging to be active due to: cramps, discomfort, lack of energy and leaking concerns | "When I'm menstruating, I don't want to go swimming. I don't want to jump up and down. I just feel weird. I just want to sit down ... I just hate it [menstruation]. I think it's disgusting.. I'm so uncomfortable." |
| Fennie (2020) (6) | South Africa | To explore how menstruation is perceived, experienced, and navigated by adolescent girls living in low-to-middle income settings in South Africa | Qualitative Study | n=48 | NA | Semi-structured focus groups  Some questions based off Adolescent Menstrual Attitude Questionnaire (AMAQ). | School | Decreased engagement in school-related PA due to menstrual symptoms (e.g. pain) and fear or exposure  Girls described being disappointed that menstruation interfered with PA Some reported a lack of understanding from schoolteachers | "I was unhappy cause I was thinking 'why must it happen now?' It's too early. Because it was too early for me. I was thinking why must it happen to me now? I still wanted to like when it’s summer then I wanna swim all the time and not worrying about periods."  "You still have to do PT [physical training] and when you have your period and they do not understand that you cannot do most of the stuff that they ask you do to, like running, otherwise it will flow more." |
| Li (2020)(7) | Australia | To identify key areas of need and explore the experiences of adolescent girls with heavy menstrual bleeding and/or dysmenorrhea | Grounded Theory | n=30 | NA | Semi-structured interviews and demographic questionnaire | Gynaecology outpatient clinic | Menstrual symptoms interfered with daily activities and led to reduced participation in sporting activities Concerns around period product usage (feeling too young to use tampons), leaking and pain when swimming  Menstruation restricting physical activity viewed negatively as not doing sport/exercise affected mood  Challenges with expressing concerns to male teachers around menstruation and sport | "I couldn't do swimming for a lot of it‚ because I had my period" "I have some mental health problems as well, and I find that sport really helps. when I'm not doing sport and then I'm on my period as well, I struggle with that because I don't have anything to put me in a positive frame of mind about it" |
| Mason (2013) (8) | Kenya | To examine the menstrual experiences of young adolescent schoolgirls | Qualitative Study | n=120 | NA | Semi-structured focus groups | School | Challenges of managing menstruation and period products lead to inability to engage in daily activities such as running, playing, and walking Challenges include not have access to products and using alternatives such as cloth, blanket, clothing and tissues. | "There are challenges because when you wear cloths, you will have to walk; you will feel that the cloth might come out before reaching your destination, so you become fearful" |
| Rajaraman (2015) (9) | India + Canada | To understand perceived benefits, facilitators, disadvantages, and barriers for physical activity among South Asian adolescents | Qualitative Study | n=61 | Focus Groups | NA | School | Social barriers: Indian girls told they are not allowed to play outdoors during menstruation Menstrual pain also described as a barrier to PA | NA |
| Schmitt (2021) (10) | USA | To explore girls’ experiences with menstruation and puberty within their families, school environments, communities, and social networks | Qualitative Study | n=78 | NA | Semi-structured interviews and Participatory group sessions | School | Social barriers: Parents/guardians dissuaded girls from using pain medication for menstrual pain and to use alternative strategies such as exercise Participation in PA (PE, sports, or dance programmes) during menstruation was described as challenging. Reasons included: menstrual pain, discomfort, fear of leaking, discomfort with uniforms, period product preference/discomfort. Girls described cultural beliefs viewing menstruation as dirty, with a perception that engaging in physical activity during school would require multiple showers that led to them refraining from PA. Girls shared that school or extracurricular sport uniforms, such as those with short or tight-fitting running shorts, created anxiety that others would observe that they were menstruating | "I was told by the head cheerleader that if you do cheerleading, you have to wear a tampon. I was not going to do that . . . so I didn't do cheerleading."  "Most of sitting out of activities is because of embarrassment. You don't want to accidentally move the wrong way and then blood smears against your shorts. You don't want someone to see the outline of your pad, you know? You just feel like you always have to close your legs" |
| Secor-Turner (2020) (11) | USA | To understand the experience of menstruation for girls in the US and its impact in the school setting | Qualitative Study | n=12 | NA | Semi-structured focus groups | School and online | Girls reported menstruation made it difficult to participate in sports and school attendance due to cramps, fear of leaking.   Girls described having to navigate period products to manage menstruation during specific sports (gymnastics). | "Sometimes my cramps are terrible...if I like planned a workout that day that wasn't required to go to like practice for our sports, I wouldn't go because I would not want to"  "I was in gymnastics practice and we had to go change into our leos [leotards] and I was in the bathroom and then I saw it and then nobody had pads so I had to put in a tampon...so I just had to figure it out." |
| Sommer (2013) (12) | Tanzania | To explore post-pubescent girls' experiences of managing their menstrual flow in school environment | Case Study | 12 Schools  n=8 interviews | Observations/In-depth Interviews | Observations/In-depth Interviews | School | Day students face walking long distances while suffering from menstrual discomfort or fatigue and inadequate materials and facilities to manage menstrual flow from the long commute/long school day. One girl highlighted the challenge for commuting girls and the benefit of dark coloured uniforms for concealing leaking. | "Girls feel uncomfortable because they feel tired, sick, cannot be active, cannot answer questions [but] because their uniform skirt is dark, they are not worried about accidents." |
| Soslashndergaard (2021) (13) | Sweden | A soma design project advocating for young adolescents to  listen to and care for their newly menstruating bodies, specifically  focusing on participation in sport | Participatory soma-based research | n=7 | 2 x 4hr Workshops | 2 x 4hr Workshops | School | Participants mentioned mood changes which affect their participation in sport activities due to getting angrier at their coaches or team members during training and there were different understandings from different people.  Participants described how menstrual pain can restrict movements due to pain intensity. However, some participants used painkillers and expressed that they actually enjoyed moving and doing sport when having menstrual cramps. | "Friends understand better than the coaches. The people we train with at school are the same age, so they experience the same as you. But the coaches don't understand, and especially because they're men. So I think that for them it's a bit difficult to talk about it. That is what I have experienced or felt when other people tried talking with them about it"  "I think it can be nice to train and move when you have menstruation. I don't know if it is because you don't think about it, but I have less pain when moving".    "Whether you have menstruation or not, you have to perform. At least I don't use it as an excuse if I have pain".   "I don't want them [the coaches] to think... for they don't understand because they don’t go through it, so they cannot get that it f***ing hurts. So I don't t want to say, 'I have menstrual pain' or 'I have pain in my stomach', but it does hurt".  "You have to perform because you also do it for other people - the team, your friends - and not just for your own sake. In team sport, you lift each other up. The responsibility is not just on yourself. If there is a championship, you cannot just skip because you have menstruation". |
| Wong (2016) (14) | China | To explore self-care strategies among Chinese adolescent girls with dysmenorrhea | Qualitative Study | n=28 | NA | Semi-structured interviews | School | Dysmenorrhea led to girls avoiding PA such as cycling and running Felt exercise increased pain severity and blood flow.  Any exercise completed during menstruation was at a slower pace | NA |

**Abbreviations**: PE = Physical Education, PA = Physical Activity, UK = United Kingdom, Yr = Year

References

1. Brown N, Forrest LJ, Williams R, Piasecki J, Bruinvels G. 'Everyone needs to be educated': pupils' voices on menstrual education. Reprod Health. 2024;21(1):121.

2. Chang YT, Chen YC, Hayter M, Lin ML. Menstrual and menarche experience among pubescent female students in Taiwan: Implications for health education and promotion practice. Journal of Clinical Nursing. 2009;18(14):2040-8.

3. Cherenack EM, Rubli J, Melara A, Ezaldein N, King A, Alcaide ML, et al. Adolescent girls’ descriptions of dysmenorrhea and barriers to dysmenorrhea management in Moshi, Tanzania: A qualitative study. PLOS Global Public Health. 2023;3(7).

4. Dolan CS, Ryus CR, Dopson S, Montgomery P, Scott L. A BLIND SPOT IN GIRLS' EDUCATION: MENARCHE AND ITS WEBS OF EXCLUSION IN GHANA. Journal of International Development. 2014;26(5):643-57.

5. Dwyer JJ, Allison KR, Goldenberg ER, Fein AJ, Yoshida KK, Boutilier MA. Adolescent girls' perceived barriers to participation in physical activity. Adolescence. 2006;41(161):75-89.

6. Fennie T, Moletsane M, Padmanabhanunni A. Adolescents' experiences of menarche and menstruation in disadvantaged schools in South Africa: a qualitative exploration. Health Education. 2021;121(4):408-19.

7. Li AD, Bellis EK, Girling JE, Jayasinghe YL, Grover SR, Marino JL, et al. Unmet Needs and Experiences of Adolescent Girls with Heavy Menstrual Bleeding and Dysmenorrhea: A Qualitative Study. Journal of Pediatric and Adolescent Gynecology. 2020;33(3):278-84.

8. Mason L, Nyothach E, Alexander K, Odhiambo FO, Eleveld A, Vulule J, et al. 'We keep it secret so no one should know' - A qualitative study to explore young schoolgirls attitudes and experiences with menstruation in rural Western Kenya. PLoS ONE. 2013;8(11).

9. Rajaraman D, Correa N, Punthakee Z, Lear SA, Jayachitra KG, Vaz M, et al. Perceived Benefits, Facilitators, Disadvantages, and Barriers for Physical Activity Amongst South Asian Adolescents in India and Canada. Journal of Physical Activity & Health. 2015;12(7):931-41.

10. Schmitt ML, Hagstrom C, Nowara A, Gruer C, Adenu-Mensah NE, Keeley K, et al. The intersection of menstruation, school and family: Experiences of girls growing up in urban areas in the U.S.A. International Journal of Adolescence and Youth. 2021;26(1):94-109.

11. Secor-Turner M, Huseth-Zosel A, Ostlund R. Menstruation Experiences of Middle and High School Students in the Midwest: A Pilot Study. Journal of School Nursing. 2020.

12. Sommer M. Structural factors influencing menstruating school girls' health and well-being in Tanzania. Compare. 2013;43(3):323-45.

13. Soslashndergaard MLJ, Ciolfi Felice M, Balaam M. Designing Menstrual Technologies with Adolescents2021. 260 (14 pp.)- (14 pp.) p.

14. Wong CL, Ip WY, Lam LW. Self-Care Strategies among Chinese Adolescent Girls with Dysmenorrhea: A Qualitative Study. Pain Manag Nurs. 2016;17(4):262-71.
