# Supplementary material for: Adolescents, menstruation, and physical activity: insights from a global scoping review
Source: BMC Womens Health. 2025 Jun 6;25:281. doi: 10.1186/s12905-025-03825-w (PMC12142975; doi:10.1186/s12905-025-03825-w)
Supplement: Supplementary file 5 — Additional file 5. Intervention studies included in review. Table of fifteen intervention studies included in the scoping review. Table displaying summary and description of intervention studies included in the scoping review. [file 12905_2025_3825_MOESM5_ESM.docx]

Table of fifteen Intervention studies included in scoping review

| Author/Year | Country | Aim/Purpose | Research Design | Sample/Population | Measure of PA | Measures of Menstruation | Context | Main Findings | Intervention |
| --- | --- | --- | --- | --- | --- | --- | --- | --- | --- |
| Abbaspour (2006) (1) | Iran | To examine the effect of exercise on primary dysmenorrhea in high school girls | RCT | n=142 1 exercise group (n=97) 1 control group (n=45) | Intervention | VAS for pain Daily Diary | School | Mean severity of pain decreased in the intervention group but no change in severity of pain in the control group.  Mean duration of pain decreased in the exercise group but not in the control group (p<0.01).  Mean duration of bleeding in exercise group decreased in the intervention group. | 1 x exercise group - 4 activities done (stretching, mobility, light intensity) twice a day but not during menstruation for 2 menstrual cycles  1 x control group |
| Abdelrahman (2024)(2) | Egypt | To compare the effects of aquatic exercise versus aerobic exercise on primary dysmenorrhea and QOL in adolescent females | RCT | n=60  1 aquatic exercise group  1 aerobic exercise group | Intervention | Questionnaire | Hospital | Both groups, A and B, experienced significant reductions in the WaLIDD  score, NPRS, and all domains of EQ‐5D‐3L (p < 0.05), coupled with significant in-  creases in PPT and EQ‐VAS (p < 0.05).  No differences between groups. Suggesting that aquatic and aerobic exercise are equally effective for decreasing dysmenorrhea severity and pain in adolescents with primary dysmenorrhea | Group A - Aquatic exercise for 12 weeks  (Aquatic exercise 3 x a week for 12 weeks, consisting of walking in water warm up, 20 min aerobic and strength exercises in the water)  Group B - Aerobic exercise for 12 weeks  (40-min aerobic treadmill sessions 3 days a week for 12 weeks) |
| Chaudhari (2013) (3) | India | 1. To estimate the prevalence of primary dysmenorrhea among schoolgirls of Chandigarh. 2. To compare the impact of exercise and hot water bottle on the occurrence and severity of primary dysmenorrhea | RCT | n=53 in PA group  n=75 in hot water bottle group (no control) | Intervention  Daily diary | Questionnaire VAS for pain Daily Diary | School | Findings suggest that exercise (and hot water bottle) led to a significant improvement in severity of pain and menstrual distress. | Group 1 demonstrated 5 exercises to be practised throughout the month except on days of menstruation, twice a day for 10-15 mins for 3 months  Group 2 told to use hot water bottle. Both groups given information on menstrual cycle and dysmenorrhea |
| Djalalinia (2012) (4) | Iran | To compare the effects of different training methods on the adolescents' reproductive health promotion for designing and implementing the most proper reproductive health training program | RCT | n=1231 1 group trained by school health trainers 1 group trained by parents  1 control group | Questionnaire | Questionnaire | School | Exercise during menstruation reported by 36.4% of participants and most of these were in the school health trainers trained group (p<0.05) | 2-year community-based health education intervention with 3 year follow-up One group trained by school health trainers (trainers trained by project experts) One group trained by parents (parents trained by project experts) Control group |
| Fallah (2018) (5) | Iran | To evaluate the effect of 3 exercise programs on reducing symptoms of primary dysmenorrhea | RCT | n=78 1 massage group 1 stretching group 1 combination group 1 control group | Intervention | Questionnaire VAS for pain | School | All 3 intervention groups found a significant reduction in severity of dysmenorrhea pain, VAS, present pain intensity and total pain (p<0.05). | 8 weeks PA intervention, 3 sessions a week and twice a day 4 groups: Massage group (10 mins each session) Stretching group (20 mins each session) Combination group (20 minutes each session) Control group (no exercise) |
| Golub (1958) (6) | USA | To explore how two therapeutic exercises impact dysmenorrhea symptoms | Non-randomised Intervention Study | n=5324 Billig and Mosher exercise group Golub exercise group | Intervention | Questionnaire | School | 74% of participants reported being 'cured' of dysmenorrhea or have 'improved' symptoms after doing the Billig exercises and 76% for the Mosher exercises. (Focus is on 6 schools as there were at least 20 girls in each group of both treatments in these schools). | Two Techniques Mosher - stimulation of circulation and relief of pelvic and leg congestion. Performed twice a day Billig - Series of stretching exercises, performed three times daily  Participants asked to do exercises for 6 months to 2 years Study ran for 8 years |
| Golub (1960) (7) | USA | To compare exercise regimens which have been suggested as therapy for dysmenorrhea | Non-randomised Intervention Study | n=942 Exercise group (n=141) Body-building Exercise group (n=61) Billig and Mosher exercise Group (n=740) | Intervention | Questionnaire | School | 35% of the exercise group reported improved dysmenorrhea symptoms and 21% of the control group reported improvements. However, difference between groups was not statistically significant.  Authors reported that Golub exercises for dysmenorrhea were similar in effectiveness to the Billig and Mosher exercise in the comparison study however it is not possible to draw conclusions. Concluded that any regular exercise involving considerable bending and twisting would be effective in reducing dysmenorrhea symptoms. | Group 1 - Conducted Golub exercise during school PE hours, twice during each gym period, 4 times a week Group 2 - Conducted Bilig and Mosher (see below for details) exercises 3 x a day for 3-7 months Control Group - Did routine body-building exercises (Golub exercise: trunk twisting and bending and extension) |
| Golub (1968) (8) | USA | To determine the impact of Golub exercises on dysmenorrhea | Non-randomised Intervention Study | n=171 1 exercise group 2 control groups | Intervention | Questionnaire | School | Girls who did not carry out the exercises developed significantly more dysmenorrhea than those who exercised The differences between the intervention and control group for dysmenorrhea symptoms were significant (p<0.05) where only 39% of the intervention group reported suffering from dysmenorrhea compared to 61% of the control group. | Golub Exercises (therapeutic exercises designed for dysmenorrhea relief) Girls were asked to perform these 3 x a day 1 control group originally but 1 additional control group formed by girls who did not want to exercise |
| Golub  (1963)(9) | USA | To evaluate the first component of a therapeutic exercise (Golub) for dysmenorrhea | Non-randomised Intervention Study | n=197  (no control) | Intervention | Teacher interview | School | 96% of girls reported improvements in dysmenorrhea  Authors concluded that the first component of the Golub exercise is at least as effective as the Billig, Mosher and complete Golub exercise (involving 2 components), possibly more effective. Authors suggest this is because of its simplicity and ease of performance which means it’s performed more diligently. | First component of the Golub exercise - twisting/bending exercises which involve no wall space and minimal floor space  Performed 3 times daily building up to 10 times for 3 months |
| Kansiime, C Hytti, L (2020) (10) | Uganda | To pilot test an intervention to improve menstrual health and hygiene and school attendance | Pilot Intervention Study | n=369 (no control) | Questionnaire | Questionnaire Qualitative Interview | School | Quantitative: reduction in the proportion of girls reporting avoiding physical activity during menstruation (from 47.8% to 25.7%, Adjusted prevalence ratio =0.55, 95% CI 0.42 to 0.71, p<0.001). Qualitative: The interviews found that non-pharmacological methods of pain relief were popular and effective "They taught us how to do exercises to relieve pain and it worked for me so, the last time, I didn't use painkillers; I managed my periods by doing exercises and using reusable pads" | Multi-component school-based menstrual health and hygiene intervention for 9 months: Training teachers to improve delivery of government guidelines for puberty education. Training in use of menstrual kit and pain management, a drama skit, provision of analgesics and improvements to school water and sanitation hygiene facilities. |
| Parkhad Suchitra (11) (2013) | India | To study the effect of yoga on premenstrual and menstrual cycle changes in girls with menstrual cycle disorders | Prospective Intervention Study (non-randomised) | n=200 (1 group, no control) | Intervention | Questionnaire | School | Participants showed reduction in dysmenorrhea, backache, lower limb pain, irritability and lack of concentration after 6 months of yoga training. (reported as significant but no p value reported) | 6 months of yoga training |
| Ramaiah (2021) (12) | Saudi Arabia | To explore the effectiveness of pelvic rocking exercises and home workouts on dysmenorrhea | RCT | n=180 Relaxation technique + hot water bottle group Mild aerobic exercise +hot water bottle group Control group | Intervention | Questionnaire | Community | Concluded that 12 weeks of either intervention significantly minimised the severity of menstrual pain compared to the control group (no statistics presented though) No statistically significant differences between 2 intervention groups so both equally effective. | Group 1- Progressive relaxation techniques (pelvic rocking and hot water application) Group 2 - Home workouts for 45 minutes (mild aerobic exercise) with hot water application Control group  Intervention for 12 weeks |
| Sefrizon, Deharnita (13) (2018) | Indonesia | To look at the effectiveness of stretching exercises and Gymnastics on Decreasing Menstrual Pain | Quasi-experimental study (non-randomised) | n=33 (no control) | Intervention | Questionnaire | School | Results showed that the exercises reduced the severity of menstrual pain from severe/moderate to moderate/mild pain. | Stretches and gymnastics exercises designed to help stretch the abdominal muscles, pelvis and waist to be done for 3 days before menstruation for 30 minutes a day |
| Shahr-jerdy (2012) (14) | Iran | To assess the effect of one term of stretching exercise on primary dysmenorrhea in high school students | RCT | n= 179 n=124 intervention group n=55 control group | Intervention | Questionnaire VAS for pain | School | Performing 8 weeks of selected stretching exercises reduces pain intensity, diminishes pain duration, and decreases the consumption of analgesics drugs in students with moderate-to-severe primary dysmenorrhea during the menstruation cycle | 1 Control group 1 Exercise group - active stretching exercises for 8 weeks (3 days per week, 2 times per day, 10 minutes each time) and to avoid performing stretching exercises during the period itself. |
| Widyanata (2017) (15) | Bali | To analyse the effectiveness of physical activity and meditation on primary dysmenorrhea | Quasi-experimental study (non-randomised) | n=40 20 in PA group 20 in meditation group (No control) | Intervention | Questionnaire VAS for pain | School | In both groups, menstrual pain decreased post intervention. The % of participants reporting moderate menstrual pain before intervention was 80% and was 20% for mild pain. This changed to all (100%) reporting mild pain post PA intervention.  No difference between PA and meditation groups | Group 1 - Physical activity intervention (PA not specified) Group 2 - Meditation treatment  Intervention done twice a week for 2 months |

**Abbreviations**: PA=Physical Activity, VAS = Visual Analogue Score, RCT = Randomised Control Trial, WaLIDD = A working ability, location, intensity, days of pain, dysmenorrhea, NPRS = Numeric Pain Rating Scale, PPT = Pressure pain threshold

References

1. Abbaspour Z, Rostami M, Najjar S. The Effect of Exercise on Primary Dysmenorrhea. Journal of Research in Health Sciences. 2006;6(1):26-31.

2. Abdelrahman AY, El‐Kosery SM, Abbassy AH, Botla AM. Effect of aquatic exercise versus aerobic exercise on primary dysmenorrhea and quality of life in adolescent females: A randomized controlled trial. Physiotherapy Research International. 2024;29(3):1-9.

3. Chaudhuri A, Singh A, Dhaliwal L. A randomised controlled trial of exercise and hot water bottle in the management of dysmenorrhoea in school girls of Chandigarh, India. Indian J Physiol Pharmacol. 2013;57(2):114-22.

4. Djalalinia S, Tehrani FR, Afzali HM, Hejazi F, Peykari N. Parents or school health trainers, which of them is appropriate for menstrual health education? International Journal of Preventive Medicine. 2012;3(9):622-7.

5. Fallah F, Mirfeizi M. How Is the Quality and Quantity of Primary Dysmenorrhea Affected by Physical Exercises? A Study Among Iranian Students. International Journal of Womens Health and Reproduction Sciences. 2018;6(1):60-6.

6. Golub LJ, Lang WR, Menduke H, Brown JO. Therapeutic exercises for teen-age dysmenorrhea; a statistical comparison of the Billing and Mosher techniques in the Philadelphia School System. Am J Obstet Gynecol. 1958;76(3):670-4.

7. Golub LJ, Menduke H, Lang WR. Further evaluation of a therapeutic exercise for teen-age dysmenorrhea. Obstet Gynecol. 1960;16:469-71.

8. Golub LJ, Menduke H, Lang WR. Exercise and dysmenorrhea in young teenagers: A 3-year study. Obstetrics & Gynecology. 1968;32:508-11.

9. Golub LJ, Menduke H, Lang WR. ONE COMPONENT OF AN EXERCISE FOR DYSMENORRHEA: TWISTING WITH BENDING. Obstet Gynecol. 1963;22:324-6.

10. Kansiime C, Hytti L, Nalugya R, Nakuya K, Namirembe P, Nakalema S, et al. Menstrual health intervention and school attendance in Uganda (MENISCUS-2): A pilot intervention study. BMJ Open. 2020;10(2).

11. Parkhad Suchitra B, Palve Sachin B, Latti RG, Kulkarni NB. Effect of yoga on premenstrual and menstrual cycle disorders in adolescent girls. Biomedicine (India). 2013;33(2):170-5.

12. Ramaiah P, Albokhary AA. Muscle Relaxation Strategies on Dysmenorrhea: An Interventional Study. Journal of Pharmaceutical Research International. 2021;33(25A):79-85.

13. Deharnita, Sefrizon. The Effectiveness of Stretch Exercise Cat Combination Techniques with Dismenore Sennes on Haid Pain Decrease in Adolescent in N 1 High School of Solok City In 2018. Pakistan Journal of Medical & Health Sciences. 2020;14(2):1494-8.

14. Shahr-jerdy S, Hosseini RS, Eivazi MG. Effects of stretching exercises on primary dysmenorrhea in adolescent girls. Biomedical Human Kinetics. 2012;4:127-32.

15. Widyanata KAJ, Putra IGY, Daryaswanti PI, editors. Physical Activity and Meditation to Reduce Primary Dysmenorrhea in Adolescent. 8th International Nursing Conference on Education, Practice and Research Development in Nursing (INC); 2017 2017

Apr 08-09; Surabaya, INDONESIA2017.
