# Supplementary material for: Adolescents, menstruation, and physical activity: insights from a global scoping review
Source: BMC Womens Health. 2025 Jun 6;25:281. doi: 10.1186/s12905-025-03825-w (PMC12142975; doi:10.1186/s12905-025-03825-w)
Supplement: Supplementary file 6 — Additional file 6. Observational studies included in review. Table of forty-three observational studies included in the scoping review. Table displaying summary and description of observational studies included in the scoping review. [file 12905_2025_3825_MOESM6_ESM.docx]

Table of fifty-four observational studies included in scoping review

| Author/Year | Country | Aim/Purpose | Research Design | Sample | Measure of PA | Measures of Menstruation | Context | Main Findings |
| --- | --- | --- | --- | --- | --- | --- | --- | --- |
| Acheampong (2019)(1) | Ghana | To provide cross-sectional data on the prevalence and predictors of dysmenorrhea, its effect, and coping mechanisms | Cross-sectional Study | n=680 | Not measured  (Participants asked impact of menstruation on daily activities in Q) | Questionnaire | School | 22.5% of participants with dysmenorrhea reported that pain restricts PAs during menstruation.  Restriction of PA was significantly associated with self-reported dysmenorrhea (p<0.04).  14.9% of participants reported using physical exercise to lessen pain. |
| Alsamti (2019)(2) | Saudi Arabia | To evaluate the prevalence, impact and management of dysmenorrhea | Cross-sectional Study | n=400 | Interview-administered questionnaire (frequency of PA) | Interview-administered Questionnaire VAS for pain | School | 1. Light PA was a stronger predictor of dysmenorrhea where those who did light-intensity PA were 7 times more likely to have dysmenorrhea compared to those who did moderate and vigorous PA (OR = 7.69, 95% CI = 0.7-79.5). |
| Armour (2020)(3) | Australia | To explore the prevalence and educational impact of pelvic and menstrual pain | Cross-sectional Study | n=2421 | Not measured  (Participants asked impact of menstruation on daily activities in Q) | Questionnaire | Online through social media | 1. 48% of respondents reported that sports and PA were impacted due to menstruation 2. Higher pain scores were positively correlated with more frequently having to miss sports, physical education or physical activities due to menstruation [rs(2419)50.335, p<.001]. |
| Banikarim (2000)(4) | USA | To determine the prevalence, management, and impact of dysmenorrhea on school attendance, academic performance, and social and sports activities | Cross-sectional Study | n=740 | Not measured  (Participants asked impact of dysmenorrhea on sports in Q) | Questionnaire VAS for pain | School | 1. 51% of participants reported that dysmenorrhea limited sports participation 2. A greater proportion of participants with severe menstrual pain reported limited sports participation compared to those with mild menstrual pain (OR 2.2, 95% CI 1.3-4.0, p<0.01).  3. 15% of participants with dysmenorrhea reported using exercise as a treatment to relieve symptoms. |
| Campbell (1999) (5) | Canada | To gain a better understanding of the use and perceived effectiveness of non-pharmacologic methods in the management of menstrual discomfort | Cross-sectional Study | n=289 | Not measured  (Participants asked if exercise is used as a coping mechanism in Q) | Menstrual Distress Management Questionnaire and Symptom Severity Scale | School | 57% of participants used exercise to manage symptoms. Exercise was perceived as greater than 50% effective in relieving menstrual symptoms. |
| Chauhan (2021)(6) | India | To examine the association of lifestyle factors such as depressive symptoms, physical activity, and substance use with menstrual problems in adolescence and explore the treatment-seeking behaviour for menstrual disorders | Cross-sectional Study | n=12707 | Interview-administered questionnaire (PA Yes/No) | Interview-administered questionnaire | Population based survey | Evidence to suggest adolescent girls who were physically inactive were 0.06 times more likely to report menstrual problems compared to those who were physically active (beta 0.06, CI -0.036-0.48). |
| Cholbeigi (2022)(7) | Iran | To investigate the relationship between a health-promoting lifestyle and menstrual pain intensity and distress among adolescent girls in Qazvin | Cross-sectional Study | n=986 | Health-promoting life profile Questionnaire | Online Survey including =  VAS, Andresh Milsom Scale for dysmenorrhea severity, Moos Menstrual Distress Q for menstrual distress | School | 42.7% experience dysmenorrhea  Exercise effected severity of dysmenorrhea pain (B=0.017, p<0.001)  Exercise has a significant effect on the severity of dysmenorrhea where severity of pain was lower among participants with higher scores of exercise |
| Curry (2023) (8) | Australia | To better understand school-based curriculum and menstrual health education in Australian schools. | Cross-sectional Study | n=2692 | NA | Questionnaire asking open-ended question: What was the most useful information you got in your Health and PE class about menstruation | Social media | Menstrual education provided little practical application of how to manage in everyday activities:  “No advice was given on dealing with pain or what the  process (if any) was at school for having menstrual pain taken seriously and treated as a consideration in test writing or sport class” (18 years) |
| Defert (2024)(9) | France | To assess the age of menarche among a large population of sixth-grade girls and to investigate dysmenorrhea, the control  of pain, and the impact on activities at school in adolescents from different socioeconomic groups | Cross-sectional Study | n=1712 | Interview administered questionnaire on impact of menstruation on PA | Interview administered questionnaire; Wong-Baker Faces Pain scale for menstrual symptoms | School | Between 5-44% of teenagers reported missing sports sessions during menstruation depending on the area they lived in (urban, rural etc...) indicating differences among socioeconomic groups  More severe abdominal pain was more likely to lead to missing sport.  Sport exemption was more frequent in the urban group (p = 0.003) and among girls with severe abdominal pain (p = 0.001) |
| Dudeja (2018)(10) | India | To assess knowledge and practices about menstruation in adolescent schoolgirls of an urban slum | Cross-sectional Study | n=211 | Not measured  (Participants asked impact of menstruation on daily activities in Q) | Questionnaire | School | 41.2% (n=87) of participants reported avoiding 'walking, running and cycling' during menstruation. |
| Fakri (2024)(11) | Brazil | To evaluate the clinical and nutritional profile of adolescents with dysmenorrhea | Cross-sectional Study | n=40 | Questionnaire (participants asked if they practiced PA Yes/No) | Questionnaire  VAS for pelvic pain | Hospital Outpatients | Practicing PA was less common among group with moderate to severe pain (34.3%) compared to those with no or mild pain (40%).  27.5% in the group who had moderate or severe pain reported that PA worsened menstrual pain |
| Farquhar (2009)(12) | New Zealand | To report on the experience of menstrual and reproductive health in a group of 16-year-old girls in an urban setting | Cross-sectional Study | n=75 | Questionnaire (frequency of PA) | Questionnaire VAS for pain | School | 45% of participants (n=33) reported that bleeding and pain limited physical activities such as sport frequently |
| Feeley (2024)(13) | USA | To determine the prevalence of period poverty in school-aged adolescents in Toledo, Ohio. | Cross-sectional Study | n=408 | Not measured  (Participants asked impact of period poverty on sports in Q) | Questionnaire | School | 29.9% of participants reported missing sports due to menstruation.  Reasons for missing ‘activities’ included heavy bleeding, not having products and 'other'. However, these were reasons for all activities (not just sport) and therefore it is not possible to identify the reasons for missing sport. |
| Femi-Agboola (2017)(14) | Nigeria | To determine the prevalence of dysmenorrhea, effects on school activities, and associated school absenteeism among secondary school girls in Ibadan, Nigeria. | Cross-sectional Study | n=460 | Interview-administered questionnaire | Interview-administered questionnaire | School | 1. Of those who suffered with dysmenorrhea, 19.9% reported 'always engaging' in exercise while 23.5% reported 'sometimes exercising' to relieve or prevent pain 2. 29% of participants engaged in physical exercise as a means of reducing menstrual pain. Almost all respondents that engaged in exercise said it was effective in reducing the intensity of the pain felt, although this was not statistically significant. |
| Finne (2011)(15) | Germany | To explore the association between age, puberty, body dissatisfaction, and physical activity decline in adolescents | Cross-sectional Study | n=3238 | Questionnaire (frequency of PA) | Interviewer led questions | School | Inactivity was more likely among those with irregular menstruation (OR = 1.71, 95% CI = 1.06-2.75) |
| Ghandour (2023)(16) | Israel | To examine the menstrual characteristics of adolescent girls living in Palestinian refugee camps in the West Bank of the Israeli-occupied Palestinian territory and Jordan, | Cross-sectional Study | n=2737 | Questionnaire | Questionnaire  WaLLID Scale | Community | Higher dysmenorrhea levels were associated with limited PA patterns  Girls who reported taking part in PA daily reported lower mean dysmenorrhea score.  Only 4.8% reported using exercise as a non-pharmacological treatment for pain (rest, herbal remedies, sleep were more widely reported).  33% reported engaging in at least 1hr of PA activity daily, 60% active for at least 1h once per 1-5 days, 7% were never physically active. |
| Hadjou (2022)(17) | France | To evaluate the prevalence of dysmenorrhea in adolescents in France and its impact on daily living | Cross-sectional Study | n=953 | Not measured  (Participants asked impact of menstruation on sports activities in Q) | Questionnaire | School | 77.2% of girls reported 'difficulties' in sports activities due to dysmenorrhea However in the univariate analysis, physical activity was not associated with severe dysmenorrhea  15.2% of girls reported using PA as a treatment for dysmenorrhea |
| Hennegan (2016)(18) | Uganda | To explore schoolgirls’ experience and appraisal of menstrual absorbents in rural Uganda | Cross-sectional Study | n=205 | Not measured  (Participants asked impact of menstruation on daily activities in Q) | Questionnaire | School | Those using existing methods (including cloth, toilet paper, underwear alone) were more likely to report avoiding physical sports or exercise than those using reusable pads (OR 5.00 95% CI 1.75-14.28). |
| Hillen (1999) (19) | Australia | To explore the prevalence, Impact, and knowledge of treatment of primary dysmenorrhea | Cross-sectional Study | n=388 | Not measured  (Participants asked impact of menstruation on daily activities in Q) | Questionnaire | School | 48% of sample reported that dysmenorrhea limited their sporting activities |
| Hoppenbrouwers (2016) (20) | Belgium | To investigate the characteristics of menstrual symptoms and their impact on social activities in young girls | Cross-sectional Study | n=792 | Not measured  (Participants asked impact of menstruation on daily activities in Q) | Parker and Sneddon menstrual disorder of teenagers questionnaire | School | 60 girls reported a negative impact of menstruation on doing sports |
| Huma (2016)(21) | Pakistan | To investigate the prevalence of menstrual characteristics, its frequency, age of menarche, symptomatology and factors associated with menstrual cycle | Cross-sectional Study | n=450 | Not measured  (Participants asked impact of menstruation on PA in Q) | Questionnaire | School | 1. 61.1% of participants reported feeling hesitant to perform any physical activity during menstruation. 2. Only 38.9% of girls took part in sport and exercise during menstruation. |
| Jena (2017)(22) | India | To explore menstrual problems and health awareness of tribal adolescent schoolgirls | Cross-sectional Study | n=300 | Not measured  (Participants asked impact of menstruation on daily activities in Q) | Questionnaire | School | 44.78% of participants reported that they restricted sports activities during menstruation |
| Jeon (2014)(23) | South Korea | To examine the factors influencing dysmenorrhea among Korean middle school adolescents | Cross-sectional Study | n=572 | Questionnaire (frequency of PA) | Menstrual Distress Questionnaire | School | No evidence in this sample of an association between exercise and dysmenorrhea. |
| Kazama (2015)(24) | Japan | To determine the prevalence of and identify factors associated with dysmenorrhea | Cross-sectional Study | n=1018 | Questionnaire (frequency of PA) | Questionnaire VAS for pain | School | Higher levels of sport club activities were significantly associated with lower prevalence of severe dysmenorrhea (p=0.045) with the highest activity group having a significantly lower prevalence (OR = 0.59, 95% CI 0.35-0.99) than the 'zero' activity group.   However, sport club activity levels were not associated with overall dysmenorrhea in this sample, only severe dysmenorrhea - exercise may relieve severe dysmenorrhea. |
| Kendel (2019)(25) | USA | To characterise a population of adolescent females who presented with heavy menstrual bleeding and joint hypermobility | Cross-sectional Study | n=30 | Not measured  (Participants asked impact of HMB on PA in Q) | Menorrhagia Impact Questionnaire (MIQ) | Patients with HMB | 87% of participants reported limitations in physical activities |
| Koff (1995) (26) | USA | To examine the knowledge and attitudes of girls who regard themselves as prepared for menstruation | Cross-sectional Study | n=224 | Not measured (sport mentioned in answers to open questions) | Questionnaire | School | Participants reported they used tampons 'so you can play sports' and 'for swimming so blood doesn't get in the pool'. |
| Lghoul (2020)(27) | Morocco | To determine the prevalence of dysmenorrhea and associated factors among adolescents in public schools in Marrakesh | Cross-sectional Study | n=364 | Questionnaire (Yes or No question to being active) | Questionnaire Pictorial Blood Loss Assessment Chart | School | No evidence of an association between presence, intensity or severity of dysmenorrhea and PA participation Maybe due to the fact this is a yes or no question and not specific at all |
| Liliwati (2007)(28) | Malaysia | To determine the prevalence of dysmenorrhea, its associated factors and its effects on school activities | Cross-sectional Study | n=300 | Not measured  (Participants asked impact of menstruation on sports activities in Q) | Questionnaire | School | Mean pain score was higher (5.93 (SD 2.36)) in girls who reported being unable to participate in sports compared to those who reported doing normal activity (4.58 (SD 1.91)) (p=0.008). |
| Lim (2018)(29) | South Korea | To investigate associations between lifestyle factors and menstrual irregularities in Korean adolescents | Cross-sectional Study | n=463 | Questionnaire (frequency of PA) | Questionnaire | Online nationally representative survey | Lack of exercise (measured as walking regularity) was associated with menstrual irregularity (OR=10.42, 95% CI 2.73-39.8, p<0.001) i.e., participants who did not walk regularly were significantly more likely to experience irregular menstruation. |
| Lukindo (2022)(30) | Canada | To estimate the impact of menstrual poverty on adolescents who reside in Nova Scotia (NS), Canada | Cross-sectional Study | n=420 | Not measured  (Participants asked impact of period poverty on sports in Q) | Questionnaire (Author developed 25-item called the adolescent Menstrual Poverty Questionnaire | Social media | 40% of participants reported missing out on sports/gym class during menstruation because of lack of money for products |
| Macleod (2020)(31) | South Africa | To investigate the social and structural barriers related to menstruation across diverse schools in the Eastern Cape | Cross-sectional Study | n=1035 | Not measured  (Participants asked impact of menstruation on sports activities in Q) | Questionnaire | School | 1. 47.8% of participants reported restricting sporting activities due to menstruation Reasons for restriction: 36.4% physical symptoms, 22% fear of swimming/unwilling to swim, 17% embarrassment, 15.2% fear of discovery, 12.8% perceived inability to perform, 8.5% desire to abstain from activities, 8% perceived increase in bleeding, 5.3% management problems.  2. Participants in more resourced schools reported physical symptoms as a major reason for not participating in sporting activities. Whilst those in under-resourced schools reported management problems and fear of discovery  3. Participants in more resourced schools were more likely to restrict sporting activities when at school whilst menstruating compared to those in less resourced schools. |
| Maruf (2013)(32) | Nigeria | To explore the association of PA and adiposity indices with primary dysmenorrhea and associated menstrual pain | Cross-sectional Study | n=1383 | Questionnaire (frequency of PA) | Questionnaire | School | Study found no association between PA level and primary dysmenorrhea (PD) occurrence but found that more participants without PD engaged in PA for more than one hour daily compared to those with PD (chi2 = 11.49, p=0.001). |
| Mohamed (2012) (33) | Egypt | To examine the prevalence, determinants, impact and treatment practices of dysmenorrhea | Cross-sectional Study | n=845 | Not measured  (Participants asked impact of menstruation on sports activities in Q) | Questionnaire VAS for pain | School | 1. 50.9% of participants reported that sports participation was affected by dysmenorrhea  2. Dysmenorrhea was significantly associated with decreased sports participation. 3. A greater proportion of participants with severe dysmenorrhea (60.9%) reported limited sports participation than those with mild menstrual pain (39.2%). Those with severe pain were 2.4 times more likely to limit sports participation compared to those with mild pain (OR 2.4 95% CI 1.4-4.0, p<0.001). 4. 7% of participants with dysmenorrhea reported using exercise to relieve menstrual symptoms |
| Negi (2018)(34) | India | To investigate the prevalence of menstrual abnormalities in adolescent girls and their association with dietary and exercise pattern | Cross-sectional Study | n=470 | Questionnaire (frequency of PA) | Questionnaire | School | Dysmenorrhea and Menstrual irregularities were more frequently observed in those who did PA 3-7 days per week (63.72% and 55.44% respectively) compared to those doing <2 days per week (36.27% and 44.55%). |
| Oguz (2016)(35) | Turkey | To define the typical menstrual characteristics of adolescent girls | Cross-sectional Study | n=879 | Not measured  (Participants asked impact of menstruation on daily activities in Q) | Questionnaire | School | 59.7% of participants reported that menstruation has a serious effect on sport and exercise. |
| Parikh (2022) (36) | India | To study the knowledge and attitude regarding menstrual hygiene among adolescent girls so that it can be then  taken as a background for designing necessary interventions in the community | Cross-sectional Study | n=127 | Not measured  (Participants asked impact of menstruation on daily activities in Q) | Interview-administered questionnaire | University (<19 years old) | 21% said they avoided physical exercise during menstruation |
| Pawar (2008)(37) | USA | To examine the effect of menorrhagia (excessively heavy or prolonged menstrual blood loss) on the quality of life of adolescents | Cross-sectional Study | n=45 | Not measured  (Participants asked impact of menorrhagia on sports in Q) | Questionnaire Pictorial Blood Loss Assessment Chart | School | There was no evidence of an association between menorrhagia and sports in this sample. |
| Poureslami (2002)(38) | Iran | To assess the level of knowledge, attitudes and behaviour of female students regarding dysmenorrhea and menstrual hygiene | Cross-sectional Study | n=250 | Not measured  (Participants asked impact of menstruation on daily activities in Q) | Questionnaire | School | 1. 33% of participants avoided physical activity or even mild exercise during their menstrual period 2. 66% of participants did not believe in the effectiveness of PA to reduce menstrual pain |
| Ravi (2017)(39) | India | To assess the psychosocial impact of menstrual problems among school going adolescent girls | Cross-sectional Study | n=350 | Not measured  (Participants asked impact of menstruation on daily activities in Q) | Questionnaire | School | 1. 51.1% of girls reported being unable to do household or sports activities due to menstrual problems.  2. Girls who suffered with menstrual problems (dysmenorrhea and menorrhagia) were 10 times more likely to be unable to perform household and sports activities compared to girls without menstrual problems (OR 10.2 95% CI 3.7-19.2, p<0.001). |
| Sathish Kumar (2016)(40) | India | To determine the prevalence of dysmenorrhea and impact on daily life in adolescent girls | Cross-sectional Study | n=703 | Questionnaire (frequency of PA) | Questionnaire | School | 1. 36.5% of participants reported that dysmenorrhea affected daily physical activities.  2. Those with more severe dysmenorrhea were more likely to report that physical activities were impacted compared to those with milder dysmenorrhea (p<0.001).  3. Those who were considered regularly active were less likely to report dysmenorrhea than those who were not regularly active (p=0.03) |
| Shellasih (2010)(41) | Indonesia | To explore the factors of primary dysmenorrhea in Junior High School Students | Cross-sectional Study | n=246 | Questionnaire (Yes or No question to being active) | Questionnaire | School | 1. Results show that there is a relationship between PA and the prevalence of primary dysmenorrhea (p=0.048).  1. Larger number of those with 'low PA levels' reported suffering with dysmenorrhea (85.4%) compared to those with 'high PA levels' (74.5%) p=0.048. |
| Shinde (2016)(42) | India | To investigate adolescent menstrual problems and its relation to BMI, eating habits and physical activity | Cross-sectional Study | n=624 | Interviewer led questions (frequency of PA) | Interviewer led questions | Obstetrics and Gynaecology Department Patients | Girls who did less PA had a higher incidence of dysmenorrhea (82.77%), irregular cycles (43.61%) and menorrhagia (19.44%).   - 82.77% of girls who did less PA (<3 days a week) reported dysmenorrhea compared to 43.93% of girls who did PA daily.   - 43.61% of girls who did less PA (<3 days a week) reported an irregular cycle compared to 13.25% of girls who did PA daily.   - 19.44% of girls who did less PA (<3 days a week) reported Menorrhagia compared to 13.25% of girls who did PA daily. |
| Tanton(43) (2021) | Uganda | To understand characteristics of menstruation and examine associations with menstrual anxiety and school attendance in Ugandan secondary school students | Longitudinal Pilot Study | n=100 | Not measured  (Participants asked impact of menstruation on PA in Q) | Questionnaire | School | 1. Avoiding physical activity during menstruation was associated with being more likely to report anxiety about the next period (OR 2.49, 95% CI 1.33-4.65, p<0.01). 2. Anxiety about the next period was more common among those who disagreed that it is healthy for a girl to run, dance or cycle during her period (65.2% vs 51.4%). |
| Teperi (1989) (44) | Finland | To study the occurrence of menstrual pain and its effect on everyday life | Cross-sectional Study | n=3370 | Questionnaire (frequency and intensity of PA) | Questionnaire | Sample derived from the 1982 National Population Registry | Girls classed as having a low level of physical activity had a higher prevalence of severe menstrual pain (26%) compared to those classed as very active (19%) (p<0.01) |
| Torres (2017) (45) | Chile | To measure the impact on quality of life in Chilean adolescents with heavy menstrual bleeding (HMB) | Cross-sectional Study | n=46 | Not measured  (Participants asked impact of menstruation on daily activities in Q) | Questionnaire | Obstetrics and Gynaecology Department Patients | 80.4% of participants reported being absent from physical education due to HMB |
| Tu (2023) (46) | USA | To describe the initial menstruation  experience and to evaluate the association of premenarchal psychosocial and sensory factors with the intensity of dysmenorrhea during the period in the fourth month | Prospective Cohort | 149 time point one (before menarche)  114 time point two (post menarche) | Not measured  (Participants asked impact of dysmenorrhea on PA) | Daily Diary | Community | 48.9% reporting having trouble with PA during menses (at 4 months, increased from first period)  Participants (59%) with higher pain recalled more frequently missing PA compared to those (28%) with reporting low pain (p=0.016) |
| Udayar (2022)(47) | India | To find the prevalence of Dysmenorrhea, its risk factors, and its impact on quality of life among teenage girls residing in tribal welfare hostels | Cross-sectional Study | n=582 | Questionnaire | Questionnaire and VAS | Community | 48.8% of participants with dysmenorrhea reported restricting PA compared to those who did not experience dysmenorrhea.    No association between P and dysmenorrhea but possibly due to interpretation of responses provided, it is unclear how PA was measured |
| Vani (2013)(48) | India | To explore the prevalence of menstrual abnormalities in school going girls and their association with dietary and exercise habits | Cross-sectional Study | n=861 | Questionnaire (frequency of PA) | Questionnaire | School | No evidence of an association between PA and menstrual abnormalities (abnormal duration of flow, passage of clots, dysmenorrhea) in this sample |
| Wang (49) (2011) | USA | To define baseline ferritin values and fatigue symptoms in a population of young females with excessive menstrual blood loss, as compared to healthy controls. | Case Control Study | n=48 Cases n=102 Controls | Not measured  (Participants asked impact of menstruation on sports in Q) | Questionnaire | Cases: Participants presenting to an Adolescent Gynaecology Clinic for initial evaluation or follow-up of HMB. Controls: Participants from Sports Medicine Clinic or Adolescent Gynaecology Clinic | 79.2% of participants with HMB reported that menses affected their ability to participate in physical education or sports compared to 36.3% of controls (p<0.001) |
| Wijesiri (2013)(50) | Sri Lanka | To assess the knowledge of and attitudes towards dysmenorrhea in adolescent girls | Cross-sectional Study | n=200 | Not measured  (Participants asked impact of menstruation on daily activities in Q) | Questionnaire | School | 1. 5% of participants reported using exercise as a pain-relief method for dysmenorrhea and 3% reported using Yoga (after rest (64%), medication (55%), and heat (20%)).  2. 46% reported being physically affected by pain (assessed by asking if they participate in sports).  3. No evidence of an association between menstrual pain and physical status (participating in sports) in the sample (p=0.887). |
| Wildayani (2023) (51) | Indonesia | To find out the relationship between Physical Activity level and the incidence of dysmenorrhea in young women at SMP Negeri 16 Padang | Cross-sectional Study | n=39 | International PA Questionnaire | Questionnaire and VAS | School | Respondents who did not exercise regularly had a 4.5 times chance of experiencing dysmenorrhea compared to  respondents who did regular exercise. |
| Wilson, Emans (1984) (52) | USA | To establish the prevalence of menstrual irregularities, premenstrual syndrome, dysmenorrhea and the usual duration of menstrual flow | Prospective Study | n=327 n=167 in 1980 (Group 1) n=160 in 1981 (Group 2) | Questionnaire | Questionnaire Menstrual Calendar | School | 1. There was no significant relationship between the number of hours a girl exercised each day and dysmenorrhea, regularity of menses or duration of flow.   2. Dance was the only sport associated with irregular menses (p = 0.03) and swimmers reported more regular menses than other sports (p = 0.05). However, swimmers were more likely to report an abnormal duration of flow (p = 0.05). |
| Wong (2011)(53) | Malaysia | To determine the prevalence of dysmenorrhea, its impact, and treatment-seeking behaviour of rural adolescents | Cross-sectional Study | n=1295 | Not measured  (Participants asked impact of menstruation on daily activities in Q) | Questionnaire | School | 1. No association between reporting of dysmenorrhea and exercise levels.  2. Exercise was not a predictor of poor concentration, absenteeism, and poor school grades due to dysmenorrhea. |
| Zacharin (2010) (54) | Australia | To assess the impact of menstruation on adolescents with developmental disabilities and their families | Cross-sectional Study | n=103 | Not measured  (Participants asked impact of menstruation on daily activities in Q) | Questionnaire | Hospital outpatients | It was found in 19 participants that the degree of impact of menstruation on participation in PA was highly predictive of menstrual management options (p=0.005). Prior to menstrual management, 74% avoided PA whereas after management only 21% avoided PA. |

**Abbreviations**: PA = Physical Activity, Q = Questionnaire, OR = Odds Ratio, CI = Confidence Interval, WaLIID = working ability, location, intensity, days of pain, dysmenorrhea, VAS = Visual Analogue Scale

References

1. Acheampong K, Baffour-Awuah D, Ganu D, Appiah S, Pan X, Kaminga A, et al. Prevalence and predictors of dysmenorrhea, its effect, and coping mechanisms among adolescents in Shai Osudoku district, Ghana. Obstetrics and Gynecology International. 2019;2019.

2. Alsamti MY, Wahby IM. PREVALENCE, IMPACT AND MANAGEMENT PRACTICES OF DYSMENORRHEA AMONG FEMALE STUDENTS AT GOVERNMENTAL SECONDARY SCHOOLS IN JEDDAH, SAUDI ARABIA, 2018. Indo American Journal of Pharmaceutical Sciences. 2019;6(6):12448-57.

3. Armour M, Ferfolja T, Curry C, Hyman MS, Parry K, Chalmers KJ, et al. The Prevalence and Educational Impact of Pelvic and Menstrual Pain in Australia: A National Online Survey of 4202 Young Women Aged 13-25 Years. Journal of Pediatric and Adolescent Gynecology. 2020;33(5):511-8.

4. Banikarim C, Chacko MR, Kelder SH. Prevalence and impact of dysmenorrhea on Hispanic female adolescents. Arch Pediatr Adolesc Med. 2000;154(12):1226-9.

5. Campbell MA, McGrath PJ. Non-pharmacologic strategies used by adolescents for the management of menstrual discomfort. Clinical Journal of Pain. 1999;15(4):313-20.

6. Chauhan S, Kumar P, Patel R, Srivastava SRO, Simon DJ, Muhammad T. Association of lifestyle factors with menstrual problems and its treatment-seeking behavior among adolescent girls. Clinical Epidemiology and Global Health. 2021;12.

7. Cholbeigi E, Rezaienik S, Safari N, Lissack K, Griffiths MD, Alimoradi Z. Are health promoting lifestyles associated with pain intensity and menstrual distress among Iranian adolescent girls? BMC Pediatr. 2022;22(1):574.

8. Curry C, Ferfolja T, Holmes K, Parry K, Sherry M, Armour M. Menstrual health education in Australian schools. CURRICULUM STUDIES IN HEALTH AND PHYSICAL EDUCATION. 2023;14(2):223-36.

9. Defert C, Cousin I, Marchand ICL, Burgazzi C, Le Pabic E, Arnaud AP. Dysmenorrhea among 12-year-old teenagers from different socioeconomic backgrounds. ARCHIVES DE PEDIATRIE. 2024;31(2):141-7.

10. Dudeja P, Sindhu A, Shankar P, Gadekar T. A cross-sectional study to assess awareness about menstruation in adolescent girls of an urban slum in western Maharashtra. International Journal of Adolescent Medicine and Health. 2018;30(4).

11. Fakri L, Ganen AD, Neto GDC. Assessment of the clinical and nutritional profile of adolescents with primary dysmenorrhea. MUNDO DA SAUDE. 2024;48.

12. Farquhar CM, Roberts H Fau - Okonkwo QL, Okonkwo Ql Fau - Stewart AW, Stewart AW. A pilot survey of the impact of menstrual cycles on adolescent health. 2009(1479-828X (Electronic)).

13. Feeley M, Afon O, Gonzalez T, Gorrell C, Warner E, Matus C. Period Poverty: Surveying the Prevalence in Toledo-Area Schools. J Womens Health (Larchmt). 2024;33(5):671-7.

14. Femi-Agboola DM, Sekoni OO, Goodman OO. Dysmenorrhea and Its Effects on School Absenteeism and School Activities among Adolescents in Selected Secondary Schools in Ibadan, Nigeria. Niger Med J. 2017;58(4):143-8.

15. Finne E, Bucksch J, Lampert T, Kolip P. Age, puberty, body dissatisfaction, and physical activity decline in adolescents. Results of the German Health Interview and Examination Survey (KiGGS). International Journal of Behavioral Nutrition and Physical Activity. 2011;8(1):119.

16. Ghandour R, Hammoudeh W, Stigum H, Giacaman R, Fjeld H, Holmboe-Ottesen G. Menstrual characteristics and dysmenorrhea among Palestinian adolescent refugee camp dwellers in the West Bank and Jordan: a cross-sectional study. ARCHIVES OF PUBLIC HEALTH. 2023;81(1).

17. Hadjou OK, Jouannin A, Lavoue V, Leveque J, Esvan M, Bidet M. Prevalence of dysmenorrhea in adolescents in France: Results of a large cross-sectional study. J Gynecol Obstet Hum Reprod. 2022;51(3):102302.

18. Hennegan J, Dolan C, Wu M, Scott L, Montgomery P. Schoolgirls' experience and appraisal of menstrual absorbents in rural Uganda: a cross-sectional evaluation of reusable sanitary pads. Reprod Health. 2016;13(1):143.

19. Hillen TI, Grbavac SL, Johnston PJ, Straton JA, Keogh JM. Primary dysmenorrhea in young Western Australian women: prevalence, impact, and knowledge of treatment. J Adolesc Health. 1999;25(1):40-5.

20. Hoppenbrouwers K, Roelants M, Meuleman C, Rijkers A, Van Leeuwen K, Desoete A, et al. Characteristics of the menstrual cycle in 13-year-old Flemish girls and the impact of menstrual symptoms on social life. European Journal of Pediatrics. 2016;175(5):623-30.

21. Jan FH, Huma ZE, Tariq N, Sajjad N. MENSTRUAL SYNDROME: SEVERITY, FREQUENCY AND SYMPTOMATOLOGY IN ADOLESCENT GIRLS OF QUETTA, PAKISTAN. Journal of Experimental Biology and Agricultural Sciences. 2016;4(2):128-32.

22. Jena P, Agasti N, Andalib S, Khandelwal K. MENSTRUAL PROBLEMS AND HEALTH AWARENESS OF TRIBAL ADOLESCENT SCHOOL GIRLS OF ODISHA- A CROSS-SECTIONAL STUDY. Journal of Evolution of Medical and Dental Sciences-Jemds. 2017;6(51):3917-21.

23. Jeon GE, Cha NH, Sok SR. Factors influencing the dysmenorrhea among Korean adolescents in middle school. Journal of Physical Therapy Science. 2014;26(9):1337-43.

24. Kazama M, Maruyama K, Nakamura K. Prevalence of dysmenorrhea and its correlating lifestyle factors in Japanese female junior high school students. Tohoku J Exp Med. 2015;236(2):107-13.

25. Kendel NE, Haamid FW, Christian-Rancy M, O'Brien SH. Characterizing adolescents with heavy menstrual bleeding and generalized joint hypermobility. Pediatric Blood & Cancer. 2019;66(6).

26. Koff E, Rierdan J. Early adolescent girls’ understanding of menstruation. Women and Health. 1995;22(4):1-19.

27. Lghoul S, Loukid M, Hilali MK. Prevalence and predictors of dysmenorrhea among a population of adolescent's schoolgirls (Morocco). Saudi Journal of Biological Sciences. 2020;27(7):1737-42.

28. Liliwati I, Verna LKM, Khairani O. Dysmenorrhoea and its Effects on School Activities Among Adolescent Girls in a Rural School in Selangor, Malaysia. Medicine and Health-Kuala Lumpur. 2007;2(1):42-7.

29. Lim HS, Kim TH, Lee HH, Park YH, Lee BR, Park YJ, et al. Fast food consumption alongside socioeconomic status, stress, exercise, and sleep duration are associated with menstrual irregularities in Korean adolescents: Korea National Health and Nutrition Examination Survey 2009-2013. Asia Pac J Clin Nutr. 2018;27(5):1146-54.

30. Lukindo M, Price V, Pike M. Estimating the impact of menstrual poverty on adolescents in Nova Scotia. PAEDIATRICS & CHILD HEALTH. 2022;27(7):421-8.

31. Macleod CI, du Toit R, Paphitis S, Kelland L. Social and structural barriers related to menstruation across diverse schools in the Eastern Cape. South African Journal of Education. 2020;40(3).

32. Maruf FA, Ezenwafor NV, Moroof SO, Adeniyi AF, Okoye EC. Physical activity level and adiposity: are they associated with primary dysmenorrhea in school adolescents? 2013(1118-4841 (Print)).

33. Mohamed EM. Epidemiology of Dysmenorrhea among Adolescent Students in Assiut City, Egypt. Life Science Journal-Acta Zhengzhou University Overseas Edition. 2012;9(1):348-53.

34. Negi P, Mishra A, Lakhera P. Menstrual abnormalities and their association with lifestyle pattern in adolescent girls of Garhwal, India. J Family Med Prim Care. 2018;7(4):804-8.

35. Esen İ, Oğuz B, Serin HM. Menstrual Characteristics of Pubertal Girls: A Questionnaire-Based Study in Turkey. J Clin Res Pediatr Endocrinol. 2016;8(2):192-6.

36. Parikh V, Nagar S. Menstrual hygiene among adolescent girls studying in a university of Gujarat. JOURNAL OF FAMILY MEDICINE AND PRIMARY CARE. 2022;11(7):3607-12.

37. Pawar A, Krishnan R, Davis K, Bosma K, Kulkarni R. Perceptions about quality of life in a school-based population of adolescents with menorrhagia: Implications for adolescents with bleeding disorders. Haemophilia. 2008;14(3):579-83.

38. Poureslami M, Osati-Ashtiani F. Attitudes of female adolescents about dysmenorrhea and menstrual hygiene in Tehran suburbs. Archives of Iranian Medicine. 2002;5(4):219-24.

39. Ravi R, Shah PB, Edward S, Gopal P, Sathiyasekaran BWC. Social impact of menstrual problems among adolescent school girls in rural Tamil Nadu. Int J Adolesc Med Health. 2017;30(5).

40. Sathish Kumar K, Konjengbam S, Devi HS. Dysmenorrhea among higher secondary schoolgirls of Imphal West district, Manipur: A cross-sectional study. JMS - Journal of Medical Society. 2016;30(1):38-43.

41. Shellasih NM, Ariyanti F. Factors of primary dysmenorrhea in junior high school students in south Tangerang City, Indonesia, 2018. Journal of Public Health and Development. 2020;18(1):73-83.

42. Shinde GR, Laddad M. OVERVIEW OF ADOLESCENT MENSTRUAL PROBLEMS AND ITS RELATION TO BMI, EATING HABITS AND PHYSICAL ACTIVITY. Journal of Evolution of Medical and Dental Sciences-Jemds. 2016;5(91):6757-61.

43. Tanton C, Nakuya K, Kansiime C, Hytti L, Torondel B, Francis SC, et al. Menstrual characteristics, menstrual anxiety and school attendance among adolescents in Uganda: a longitudinal study. BMC Womens Health. 2021;21(1):410.

44. Teperi J, Rimpelä M. Menstrual pain, health and behaviour in girls. Soc Sci Med. 1989;29(2):163-9.

45. Torres R, Zajer C, Menendez M, Jose Canessa M, Cerda J, Angelica Wietstruck M, et al. Heavy menstrual bleeding affects quality of life in adolescents. Revista Chilena De Pediatria-Chile. 2017;88(6):717-22.

46. Tu FF, Hellman KM, Darnell SE, Harber KA, Bohnert AM, Singh L, et al. A multidimensional appraisal of early menstrual pain experience. Am J Obstet Gynecol. 2024;230(5):550.e1-.e10.

47. Udayar SE, Jeergiyal DP, Kruthika K. Prevalence and Predictors of Dysmenorrhea and its Impact on Quality of Life among Tribal Adolescent Girls in India. Unnes Journal of Public Health. 2022;11(1):23-32.

48. Vani KR, K SV, L S, Kumar VRH, A B. Menstrual abnormalities in school going girls - are they related to dietary and exercise pattern? J Clin Diagn Res. 2013;7(11):2537-40.

49. Wang W, Bourgeois T, Klima J, Berlan ED, Fischer AN, O'Brien SH. Iron Deficiency and Fatigue in Adolescent Females with Heavy Menstrual Bleeding. Blood. 2011;118(21):1802-.

50. Wijesiri HSMSK, Suresh TS. Knowledge and attitudes towards dysmenorrhea among adolescent girls in an urban school in Sri Lanka. Nursing and Health Sciences. 2013;15(1):58-64.

51. Wildayani D, Lestari W, Ningsih WL, Sujendri S. The Relationship Between Physical Activity Level and Dysmenorrhoea in Young Women. Medical Journal of Malaysia. 2023;78(4):495-9.

52. Wilson C, Emans SJ, Mansfield J, Podolsky C, Grace E. The relationships of calculated percent body fat, sports participation, age, and place of residence on menstrual patterns in healthy adolescent girls at an independent New England high school. J Adolesc Health Care. 1984;5(4):248-53.

53. Wong LP. Attitudes towards dysmenorrhoea, impact and treatment seeking among adolescent girls: A rural school-based survey. Australian Journal of Rural Health. 2011;19(4):218-23.

54. Zacharin M, Savasi I, Grover S. The impact of menstruation in adolescents with disabilities related to cerebral palsy. Archives of Disease in Childhood. 2010;95(7):526-30.
