# Supplementary material for: Adolescents, menstruation, and physical activity: insights from a global scoping review
Source: BMC Womens Health. 2025 Jun 6;25:281. doi: 10.1186/s12905-025-03825-w (PMC12142975; doi:10.1186/s12905-025-03825-w)
Supplement: Supplementary file 7 — Additional file 7. Mixed-methods studies included in review. Table of three mixed-methods studies included in the scoping review. Table displaying summary and description of mixed-methods studies included in the scoping review. [file 12905_2025_3825_MOESM7_ESM.docx]

Table of t1hree mixed methods studies included in scoping review

| Author/Year | Country | Aim/Purpose | Research Design | Sample/Population | Measure of PA | Measures of Menstruation | Context | Main Findings | Quotes |
| --- | --- | --- | --- | --- | --- | --- | --- | --- | --- |
| Rastogi (2019) (1) | India | To elicit the knowledge, attitudes, and practices of adolescent girls regarding issues related to menstruation and to assess the barriers that prevent them from practising menstrual hygiene | Mixed Methods Study | n=187 Questionnaire 4 Focus Groups | Not measured  (Participants asked impact of menstruation on PA in Questionnaire) + Focus groups | Questionnaire Focus Group | School | 21.9% of girls adhered to the cultural belief that it was important to cease all activities involving physical exertion during menstruation  0% of girls reported using walking as a method of coping with discomfort compared to 35.1% who used rest and 30.6% who used heat therapy among others. Likely due to the fact they were forbidden by their mothers to participate in PA during menstruation. | Girls could not understand why certain myths were perpetuated by their mothers and restrictions (such as reduced physical activities) were imposed on them during menstruation |
| Vashisht (2018) (2) | India | To evaluate the various factors associated with school absenteeism during menstruation, and to assess the practices regarding menstrual hygiene | Mixed Methods Study | n=600 Questionnaire n=120 in 20 focus groups | Not measured  (Participants asked impact of menstruation on sports in Questionnaire) + Focus groups | Questionnaire Focus Group | School | Survey: 58.5% of participants reported that they were unable to participate in sports due to menstruation | Focus Groups: Absent from school due to fear, shame and teasing and didn't want other people to find out (classmates, boys),  Inadequate sanitary products hinder attendance at school - they could not offer proper products all the time and used old cloth at home |
| Zipp (2023)(3) | Zambia | To better understand the lived experience of menstrual health amongst adolescent girls in the field of sport for development (SFD), the impact of menstrual health education through SFD and in what ways SFD might serve as a platform for menstrual health education | Mixed Methods Study | n=49 | Not measured directly,  Lesson provided on program on stretching and breathing exercises to cope with menstrual pain | Diary completed during lessons asking about period and impact on lifestyle | School | All respondents said they would try the exercises at home following the intervention. Although mixed response completing the exercises, some felt uncomfortable stretching during their period. Unclear if this was physical or from embarrassment.  Two (n=2) discovered they were leaking during a sport or PA session.  Authors suggested that encouraging PA throughout menses may dissuade people from quitting sport or skipping physical education lessons during their periods. | “I started my periods this year I did not know what it was its at school when we were playing at the ground and I was in white my one if my classmate came and told me that I had blood on my shorts, she told me to go and see the teacher I went the teacher gave me something to cover myself with I went home and told mom who later called my Grandmother. Grandmother talked to me on how a girl need to take care of herself,” (Age 12) |

Abbreviations: PA=Physical Activity

1. Rastogi S, Khanna A, Mathur P. Uncovering the challenges to menstrual health: Knowledge, attitudes and practices of adolescent girls in government schools of Delhi. Health Education Journal. 2019;78(7):839-50.

2. Vashisht A, Pathak R, Agarwalla R, Patavegar BN, Panda M. School absenteeism during menstruation amongst adolescent girls in Delhi, India. Journal of Family and Community Medicine. 2018;25(3):163-8.

3. Zipp S, Mwambwa L. Menstrual Health Education in Sport for Development: A Case Study from Zambia. JOURNAL OF SPORT FOR DEVELOPMENT. 2023;11(2).
